# Studying the distribution patterns, dynamics and influencing factors of city functional components by gradient analysis

**DOI:** 10.1038/s41598-021-97208-4

**Published:** 2021-09-07

**Authors:** Shun Liu, Guofu Yang, Zhaoping Wu, Feng Mao, Zelong Qu, Ying Ge, Jie Chang

**Affiliations:** 1 College of Life Sciences, Zhejiang University, Hangzhou, 310058 China; 2 School of Earth and Environmental Sciences, Cardiff University, Cardiff, CF10 3AT UK

**Keywords:** Ecology, Ecology, Environmental sciences, Environmental social sciences

## Abstract

Understanding the spatial distribution characteristics and formation mechanism of urban facilities (city functional components) constitutes the basis of urban layout optimization. Currently, research on the overall distribution of the various types of city functional components is lacking. In this study, by applying the gradient analysis method common in ecology, we considered 13 types of city functional components (80,214 individuals in total) in large, medium and small Chinese cities (9 cities in total) to carry out quantitative analysis of the distribution of components along urban–rural gradients through density distribution curves. The results indicated that: (1) a higher density of city functional components near the city centre revealed an obvious aggregated distribution; (2) the spatial distribution dynamics of city functional components were related to the city size, providing a reference for the rational distribution of components in cities of different sizes; (3) the distribution of city functional components was affected by their ecosystem services. This study offers a new perspective for the application of ecological methods in the examination of the distribution of city functional components.

## Introduction

With the acceleration of urbanization and changes in lifestyles, an increasing number of urban facilities will be required^1,2^. The ideal distribution of facilities could provide better services to more people^3^, reduce travel costs^4^, and improve land-use efficiency^5,6^. Understanding the spatial distribution dynamics of urban facilities and their formation mechanism comprises the basis for urban layout optimization, which is highly important.

Most studies on the distribution of urban facilities (i.e., city functional components, such as restaurants, banks, and gas stations in this study) have focused on the analysis of the distribution of a single type of component and the factors influencing its distribution. Early studies have proposed that city functional components can freely locate to maximize their profits^7^. Components with a higher rent-paying ability are located closer to the city centre^8,9^. The government’s zoning plan for regulating land use is another important factor affecting the distribution of city functional components^10,11^. For example, the number of convenience stores is low in specified areas affected by the government’s zoning regulations^12^. Due to the NIMBY (not in my back yard) effect, the public participatory zoning decisions also affect the distribution of components. Residents want “green” amenities rather than facilities with potential environmental or health impacts that are closer to their domiciles^13^. The distribution of city functional components is also influenced by many other location factors, including population, economy, consumer preferences, and agglomeration effects^14–16^. Some researchers have also shown that the factors affecting the spatial pattern of components are always multiple and intertwined^17–20^. Moreover, studies have focused on the changes in the distribution of city functional components with city development. For example, the concentration of restaurants notably increases due to the substantial customer base in central business districts and then decreases due to the emergence of new housing and retail areas^21,22^. Logistics facilities and farms constantly move to the suburbs due to conflicts with residential neighbourhoods caused by environmental pollution^23–25^. However, previous studies have focused mostly on a single type of city functional component, and studies on the overall distribution dynamics of multiple types of components are thus lacking, a situation that does not facilitate a comprehensive understanding of the distribution of city functional components.

There are many similarities between urban systems and biological communities in regard to their structure^26^, so mature theories and methods in ecology have been gradually introduced into the field of urban research. For example, the niche theory in ecology has been applied to the comparative study of the competitive advantages between enterprises^27^. Taylor’s power law commonly used in ecological studies has also been verified in the study of the spatial distribution of city functional components^28^. The relationship between the number of city functional component types and sampling area has also been demonstrated to be consistent with the saturation curve relationship between species and area in ecology^29^. Recent studies have found that the distribution of city functional components along urban–rural gradients is similar to that of biological species along ecological gradients, exhibiting monotonic or unimodal shapes^11,30,31^. In fact, existing studies have used gradient analysis to study the distribution of city functional components. For example, Beijing, China, clearly shows the zoning of functional components from the city centre to the suburbs^32^. The diversity of functional components of the case cities in China gradually decreases from the city centre to the suburbs^29^. The uneven distribution of facilities along the urban–rural gradient causes social inequality^33,34^. Gradient analysis could quantify the distribution dynamics of species and the relationship between species distribution and environmental factors through density distribution curves^35,36^. This transdisciplinary method can be used to study the distribution dynamics of various types of city functional components. It also provides technical support to reveal the relationship between components and location factors, which will clarify the patterns of urban development and the overall distribution of city functional components.

In this paper, we studied 13 types of city functional components in large, medium, and small cities (9 representative cities in China), such as fast-food restaurants (4 types), banks (1 type), sports venues (1 type), express outlets (2 types), gas stations (2 types), wastewater treatment plants (WTPs), greenhouses (GHs) and dairy farms (DFs) (80,214 individual components in total). We adopted the gradient analysis method common in ecological studies to explore the following: (1) the density distribution of each type of city functional component was analysed in concentric rings from the city centre to the suburbs; (2) characteristic values of the density distribution curve of each type of component were extracted along the urban–rural gradient including the peak value, peak position, niche width, kurtosis and skewness; (3) the dynamic changes in the distribution of the various types of city functional components were examined over time; and (4) the distribution mechanism of the city functional components was investigated along the urban–rural gradient.

## Results

### Spatial distribution of the city functional components along the urban–rural gradient

**Figure 1:**
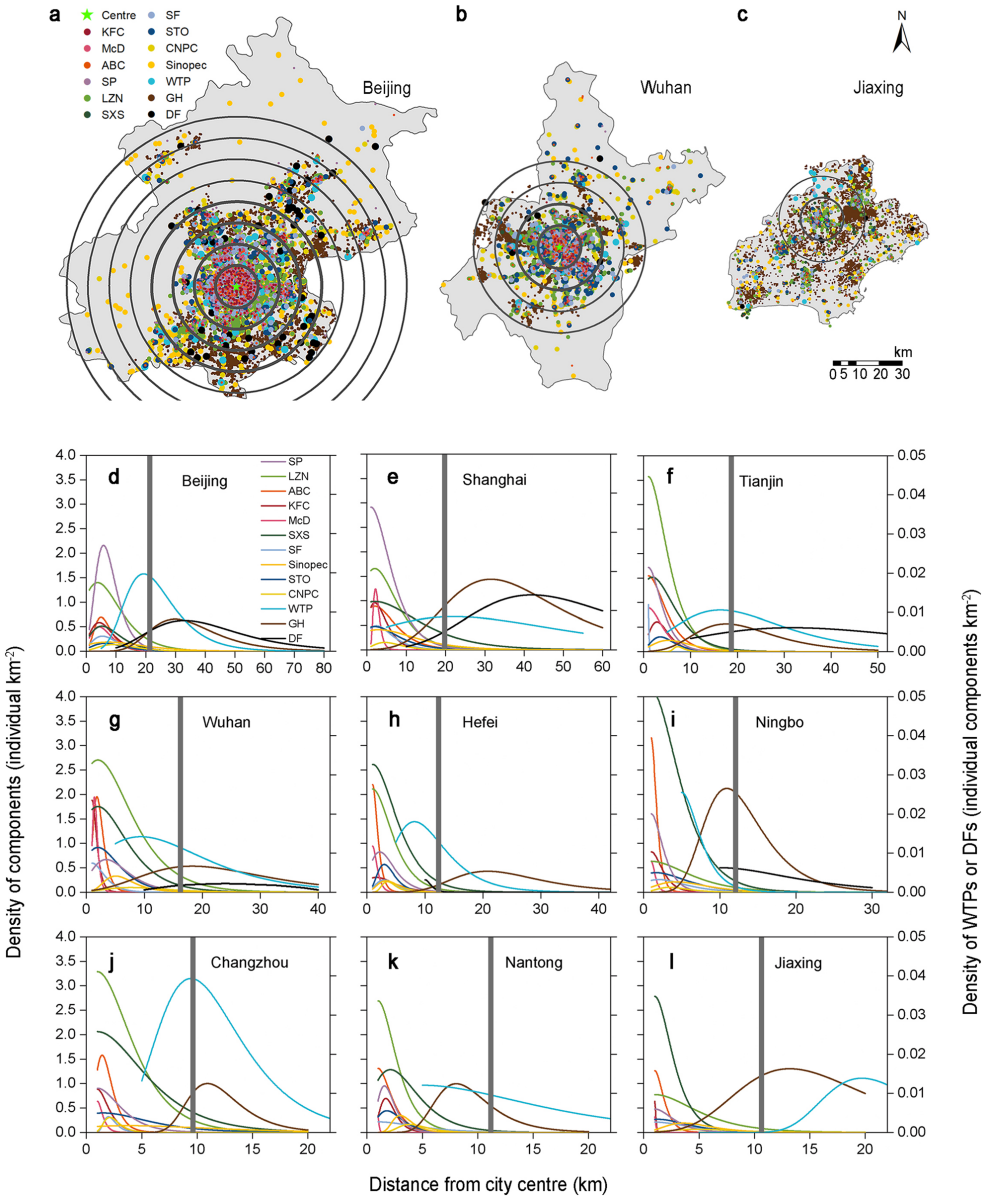
Spatial distribution of the various city functional components along urban–rural gradients. (**a–c**) Spatial point distribution of the various city functional components. The concentric rings are drawn from the city centre to the city edge, which are centred on the city centre, and the radius of the concentric rings is increased at 10-km intervals. The maps were created using ArcGIS 10.3 software (http://www.esri.com/arcgis/). (**a**) Beijing; (**b**) Wuhan; (**c**) Jiaxing. (**d–l**) Density distribution curves of the various city functional components along the urban–rural gradient. The boundary of the urban built-up area is marked with a grey band. The WTP and DF components use secondary coordinates. (**d**) Beijing; (**e**) Shanghai; (**f**) Tianjin; (**g**) Wuhan; (**h**) Hefei; (**i**) Ningbo; (**j**) Changzhou; (**k**) Nantong; (**l**) Jiaxing. The studied city functional components include KFC, McD, ABC, SP, LZN, SXS, SF, STO, CNPC, Sinopec, WTP, GH, and DF.

To better understand the spatial distribution of the city functional components along the urban–rural gradient, first, we employed a geographic information system to mark all individual functional components on a map. The location with the highest land price was defined as the city centre. We generated concentric circles with increasing radius adopting the city centre as the circle centre (Fig. 1a–c and Supplementary Fig. S1). Statistics on the number of each type of city functional component within each concentric ring were obtained to calculate the density of these city functional components. Then, the density within each ring and the distance from the edge of the ring to the city centre were fitted with the nonlinear least-squares method to acquire density distribution curves of the city functional components.

The results for the 9 case cities indicated that the peak position (d^*^) of the density distribution curves of the western fast-food restaurants (KFC and McD), banks (ABC), sports venues (SP), Chinese fast-food restaurants (LZN and SXS), express outlets (SF and STO), and gas stations (CNPC and Sinopec) occurred in built-up areas. The d* value of the WTP and GH density distribution curves occurred near the edge of built-up areas. In large cities, the d* value of the DF density distribution curve was observed in the outer suburbs outside the built-up areas (Fig. 1d–l).

### Relationship between the characteristics of the density distribution curves of the city functional components and their ecosystem services

**Figure 2:**
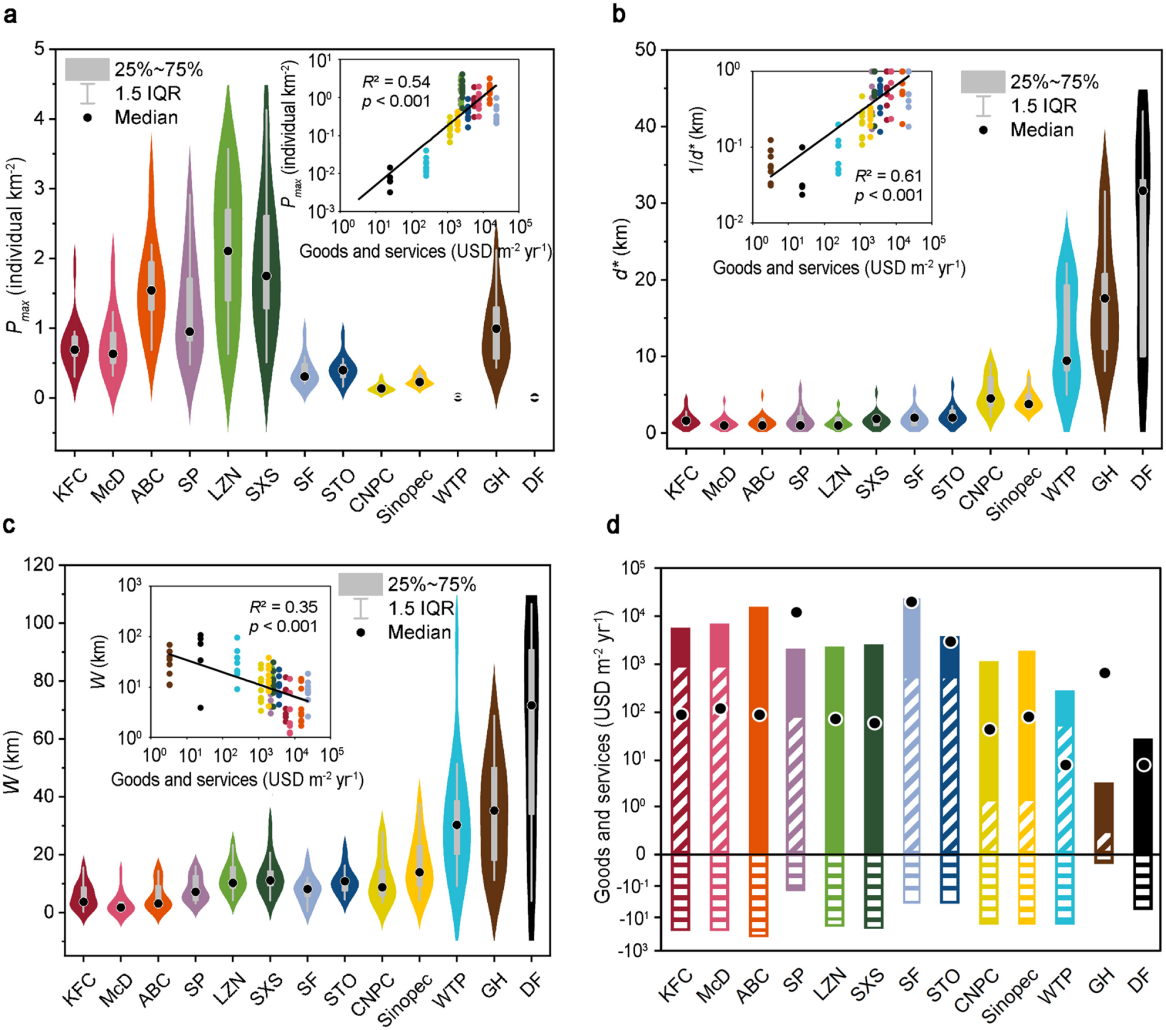
Relationship between the characteristics of the city functional component density distribution curves and ecosystem services. (**a**) *P*_*max*_ of the different types of city functional components in the case cities. The inset shows the relationship between *P*_*max*_ and the ecosystem services (the inset does not include *P*_*max*_ of the GHs because the counting method for GHs is different from that for the other types of components). (**b**) d* of the different types of city functional components in the case cities. The inset shows the relationship between 1/d* and the ecosystem services. (**c**) *W* of the different types of city functional components in the case cities. The inset shows the relationship between *W* and the ecosystem services. (**d**) Ecosystem services of the various city functional components. The solid column indicates the target services, the diagonal filled column indicates the positive accompanied services, the horizontal filled column indicates the negative accompanied services, and the black dot indicates the ecological index (the ratio of the target services to the negative accompanied services). The abbreviations of the component names are the same as those in Fig. 1.

First, we calculated statistics on the characteristic values of the density distribution curves of the various components in the 9 case cities, including the peak value (*P*_*max*_), peak position (d^*^), niche width (*W*), skewness and kurtosis. Through analysis of the *P*_*max*_ values, we found that the three types of city functional components with the highest *P*_*max*_ value included Chinese fast-food restaurants (the mean of *P*_*max*_: 2.09 individual components km^−2^ for LZN; 1.96 individual components km^−2^ for SXS) and banks (ABC, the mean *P*_*max*_ value was 1.62 individual components km^−2^), and the *P*_*max*_ value of the WTP and DF components was the lowest (Fig. 2a). Through analysis of d*, we determined that the six types of city functional components with d* values that were the closest to the city centre in the built-up areas included the western fast-food restaurants (the mean of d*: 1.53 km for McD; 1.83 km for KFC), Chinese fast-food restaurants (the mean of d*: 1.53 km for LZN; 1.91 km for SXS), banks (ABC, the mean d* value was 1.69 km) and sports venues (SP, the mean d* value was 2.00 km). Within the built-up areas, the furthest d* from the city centre was that of the gas stations (the mean of d*: 4.29 km for Sinopec; 5.15 km for CNPC). Additionally, the WTP (the mean d* value was 12.73 km) and GH (the mean d* value was 17.90 km) d* values were located at the edge of the built-up areas. The DF d* value (the mean d* value was 25.32 km) was located in the outer suburbs (Fig. 2b). Through analysis of the *W* values, we observed that the four narrowest types of city functional components were the western fast-food restaurants (the mean of *W*: 4.54 km for McD; 6.60 km for KFC), banks (ABC, the mean *W* value was 5.80 km), and sports venues (SP, the mean *W* value was 8.15 km). The *W* value of the DF component was the widest, at 61.39 km (Fig. 2c). The skewness and kurtosis results are listed in the Supplementary Table S1.

We also calculated the ecosystem services of the above 13 types of city functional components, and the results suggested that banks yielded the highest target services per unit area (15,138 USD m^−2^ yr^−1^), followed by the western fast-food restaurants (mean value for KFC and McD: 5,631 USD m^−2^ yr^−1^). The WTP and DF target services were lower (at 213 and 27 USD m^−2^ yr^−1^, respectively), while the GH target services were the lowest (2.94 USD m^−2^ yr^−1^). In addition, in ascending order, the dis-services followed the order of GHs, sports venues (SP), express outlets (SF and STO), DFs, gas stations (CNPC and Sinopec), WTPs, restaurants (KFC, McD, LZN, SXS) and banks (ABC). Among them, the ecological indexes of the express outlets, sports venues and GHs were higher, at 12,068 (the mean value for SF and STO), 12,025 and 674, respectively. However, the ecological indexes of the gas stations, DFs and WTPs were relatively low, at 60.37 (the mean value for CNPC and Sinopec), 8.16 and 7.56, respectively (Fig. 2d).

Via regression analysis of the characteristic values of the density distribution curves and ecosystem services, we found that the peak centrality (1/d*) of the density distribution curves was significantly positively correlated with the net ecosystem services. The *P*_*max*_ value of the density distribution curves was positively correlated with the net ecosystem services. The *W* value of the density distribution curves was significantly negatively correlated with the net ecosystem services (please refer to the insets in Fig. 2a–c).

### Relationship between the characteristics of the density distribution curves of the city functional components and city size

**Figure 3:**
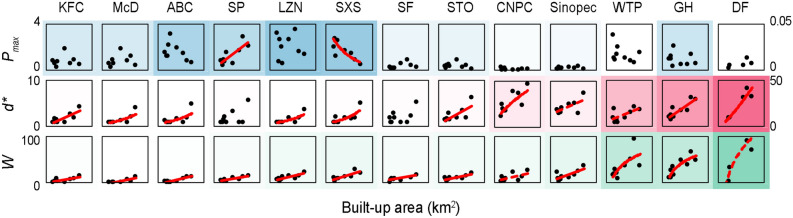
Relationship between the characteristic values (*P*_*max*_, d^*^, *W*) of the density distribution curves of the city functional components and the city size (built-up area). The WTP and DF components use secondary coordinates. The red line indicates the results of correlation analysis of the scatter points in the figure, all *p* values < 0.05. The depth of the background colour indicates the mean value of the ordinate of the scatter points in each small figure. The abbreviations of the component names are the same as those in Fig. 1.

We analysed the difference in the characteristic values [peak value (*P*_*max*_), peak position (d^*^), niche width (*W*)] of the density distribution curves for city functional components of the same type among 9 case cities. The results showed that the *P*_*max*_ of the density distribution curves of the same functional components in different cities had no significant correlation with the urban built-up area, the d^*^ was positively correlated with the built-up area, the *W* was positively correlated with the built-up area (Fig. 3). In addition, we analysed the correlation between the characteristic values (*P*_*max*_, d^*^, *W*) of the density distribution curves of city functional component and other attributes [GDP (gross domestic product) or population] of city size. The correlation results were similar to the correlation result between the characteristic values of the density distribution curves and the built-up area (Supplementary Fig. S3).

### Relationship between the characteristics of the density distribution curves of the city functional components

**Figure 4:**
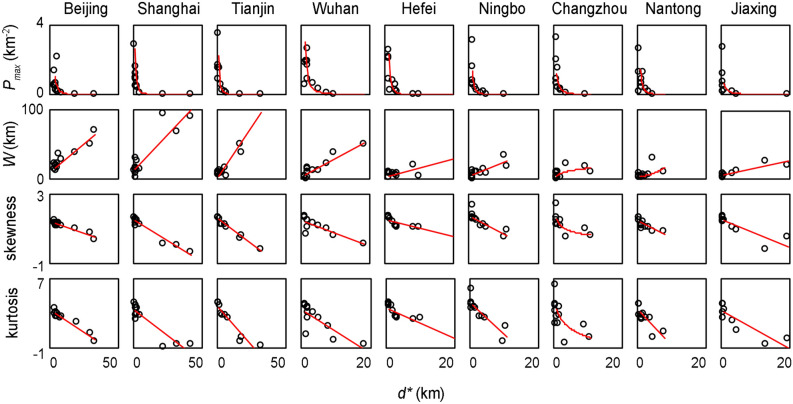
Relationship between d* and the other characteristic values (*P*_*max*_, *W*, skewness, and kurtosis) of the density distribution curves of the city functional components. The red line indicates the results of correlation analysis of the scatter points in the figure, all p-values < 0.05.

To analyse the relationship between the five characteristic values of the density distribution curves of the 13 types of city functional components [the peak value (*P*_*max*_), peak position (d^*^), niche width (*W*), skewness, and kurtosis], four characteristic values (*P*_*max*_, *W*, skewness, and kurtosis) and d* were subjected to correlation analysis. In all case cities, we drew the following conclusions: the *P*_*max*_ value of the density distribution curves of the 13 types of components and the d* value attained a significant negative correlation, *W* was positively correlated with d*, the skewness was negatively correlated with d*, and there was a significant negative correlation between the kurtosis and d* (Fig. 4). Moreover, we analysed the correlation between three characteristic values (*W*, skewness, and kurtosis) and the *P*_*max*_ value of the density distribution curves of the 13 types of city functional components in each case city and found that *W* was negatively correlated with *P*_*max*_, the skewness was positively correlated with *P*_*max*_, and there existed a positive correlation between the kurtosis and *P*_*max*_ (Supplementary Fig. S4).

### Dynamic analysis of the density distribution curves of the city functional components along the urban–rural gradient over time

**Figure 5:**
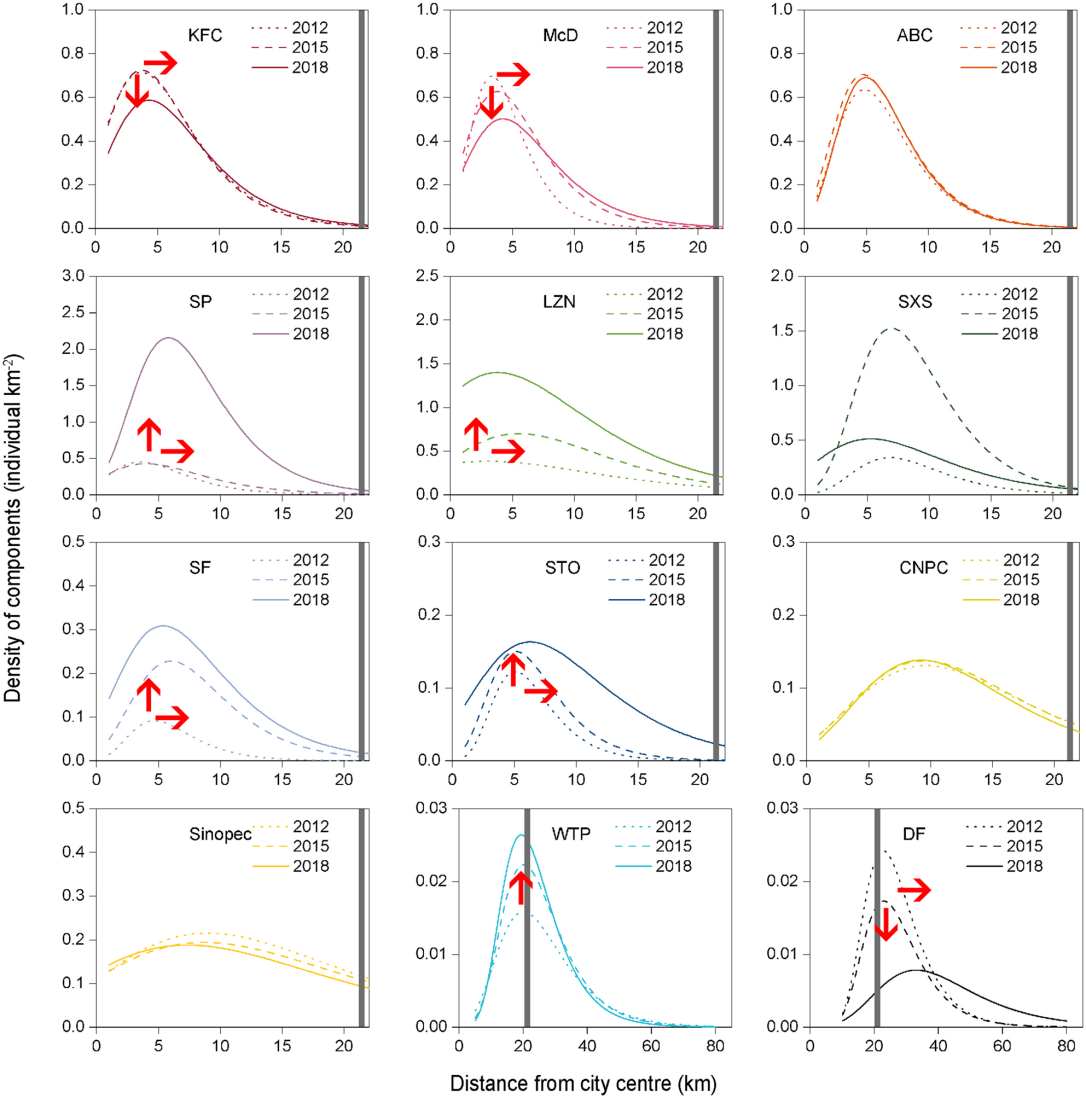
Dynamic changes in the density distribution curves of the city functional components along the urban–rural gradient over time (Beijing). The arrow indicates the change trend of the density distribution curve of the city functional components. The grey band indicates the boundary of the urban built-up area. The abbreviations of the component names are the same as those in Fig. 1.

Choosing Beijing as an example, we fitted the density distribution data of 12 types of city functional components along the urban–rural gradient in 2012, 2015 and 2018. We found that the peak value (*P*_*max*_) of the density distribution curves of the sports venues (SP), Chinese fast-food restaurants (LZN and SXS) and express outlets (SF and STO) greatly increased, i.e., from 0.45 to 2.16 individual components km^−2^ for SP, from 0.38 to 1.40 individual components km^−2^ for LZN, from 0.34 to 0.51 individual components km^−2^ for SXS, from 0.09 to 0.31 individual components km^−2^ for SF, and from 0.12 to 0.16 individual components km^−2^ for STO. Certain changes in the peak position (d^*^) and niche width (*W*) also occurred. However, the *P*_*max*_ value of the density distribution curves of the western fast-food restaurants (KFC and McD) and DFs decreased from 0.71 to 0.59 individual components km^−2^ for KFC, from 0.70 to 0.50 individual components km^−2^ for McD, and from 0.024 to 0.008 individual components km^−2^ for DF. The corresponding d* values moved significantly away from the city centre, and the *W* values increased to different degrees. The *P*_*max*_ value of the density distribution curve of the WTPs was slightly enhanced from 0.016 to 0.020 km, but its d* and *W* values did not change significantly (Fig. 5). In addition, the *P*_*max*_, d* and *W* values of the density distribution curves of the banks (ABC) and gas stations (CNPC and Sinopec) did not change significantly. Please refer to the Supplementary Table S2, for the results of the skewness and kurtosis. Please refer to the Supplementary Fig. S5, for the dynamic change results of the density distribution curves of the city functional components in the other 8 case cities.

### Spatial distribution dynamics of the city functional components in cities of different sizes

**Figure 6:**
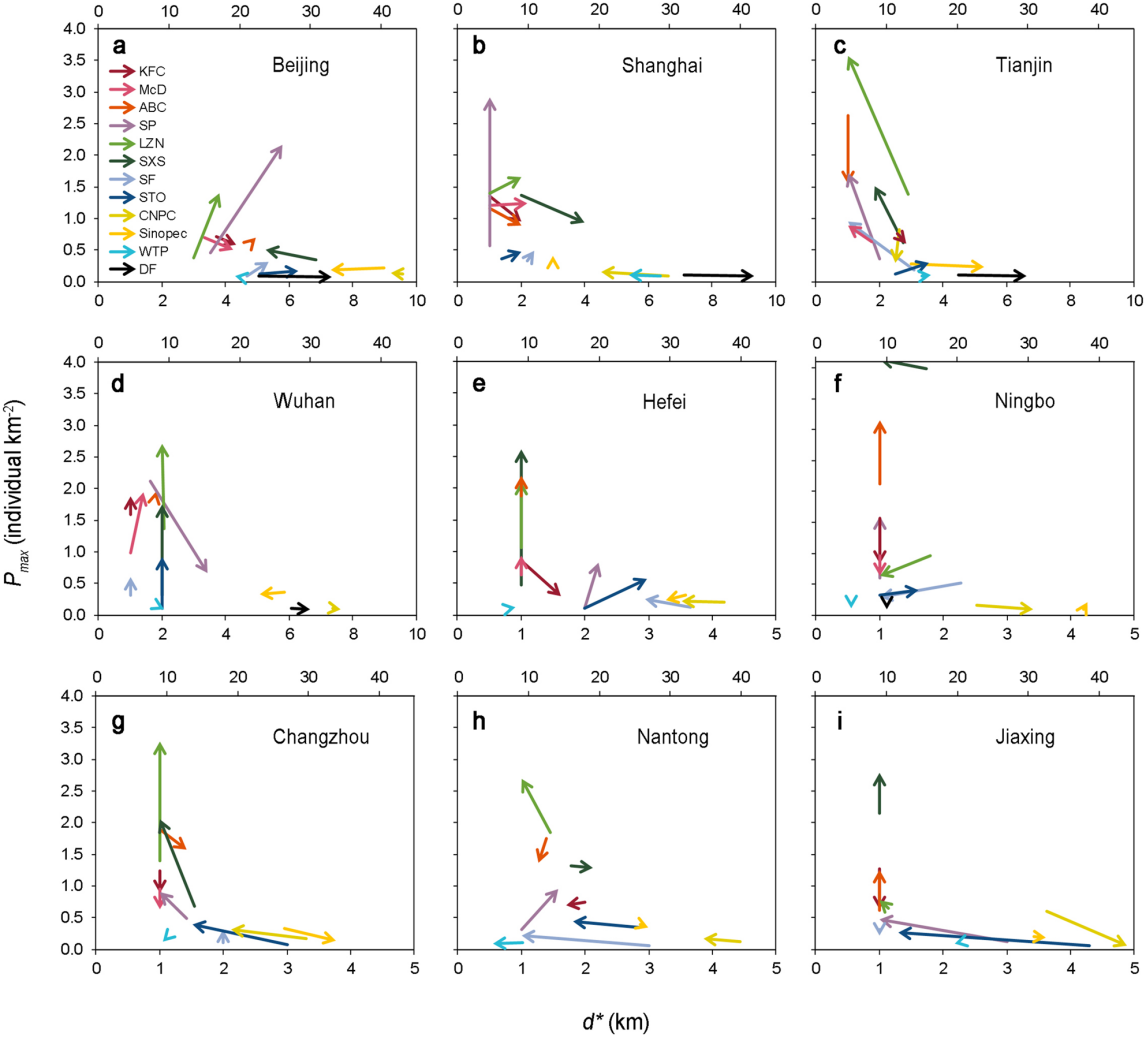
Dynamic changes in *P*_*max*_ and d* of the density distribution curves of the city functional components in cities of different sizes over time. The arrows indicate the changes in *P*_*max*_ and d*. Only the WTP and DF components use the top coordinates. (**a**) Beijing; (**b**) Shanghai; (**c**) Tianjin; (**d**) Wuhan; (**e**) Hefei; (**f**) Ningbo; (**g**) Changzhou; (**h**) Nantong; (**i**) Jiaxing. The abbreviations of the component names are the same as those in Fig. 1.

The dynamic changes in the peak value (*P*_*max*_) and peak position (d^*^) of the density distribution curves over time were further analysed to explore the differences in the distribution dynamics of the city functional components between cities of varying sizes. The results demonstrated that the *P*_*max*_ value of the sports venues (SP), Chinese fast-food restaurants (LZN and SXS) and express outlets (SF and STO) significantly increased in most cities from 2012 to 2018. In large cities such as Beijing and Shanghai, the d* value of the western fast-food restaurants (KFC and McD), Chinese fast-food restaurants and express outlets all moved away from the city centre (except SXS in Beijing). The d* value of the express outlets in three small cities, namely, Changzhou, Nantong and Jiaxing, moved from a remote location to a location at a distance ranging from 1–2 km to the city centre over time. In Beijing, Shanghai, Tianjin, and Wuhan, the d* value of the DFs moved away from the city centre. However, the d* value of city functional components such as banks (ABC), gas stations (CNPC and Sinopec) and WTPs in most case cities revealed no obvious movement (Fig. 6).

## Discussion

In natural ecosystems, species response curves along ecological gradients are usually symmetric, skewed or monotonic^35–37^. We found that the density distribution curves of the various city functional components in the urban system also exhibited these three shapes (Fig. 1d–l). There existed significant correlations between the characteristic values of the density distribution curves of the city functional components along urban–rural gradients in the 9 case cities of different scales. The niche width (*W*) and peak position (d^*^) of the density distribution curves of the city functional components revealed a significant positive correlation (Fig. 4), the closer to the city centre, the narrower the niche width of the components is. This pattern is similar to the regularity in natural ecosystems, which indicates that a higher resource level enhances the abundance of species, which causes more intense competition. Therefore, the niche of a given species is narrower^38,39^. But in the urban system, the high agglomeration of service industries in the city centre may be related to active planning, which is beneficial in reducing transport costs and the related infrastructure loads and increasing accessibility for people^4,16^. The skewness and peak position (d^*^) of the density distribution curves of the city functional components indicated a significant negative correlation (Fig. 4), which is consistent with the significant negative correlation between the niche skewness and peak position of species in natural ecosystems along ecological gradients^40^. The closer the peak position of the species or component distribution curve occurs to a high resource level, the higher the skewness of the curve towards the resource centre. Our research quantified the niche characteristics of the city functional components along urban–rural gradients and verified the applicability of ecological gradient analysis in the study of urban facility distribution.

In recent years, studies have reported that soft location factors (such as consumer preferences, laws and regulations) also play an important role in the distribution of city functional components^13,17,19^. In this study, we considered the concept of ecosystem services to quantify soft location factors such as environmental impact and human preferences. With regression analysis, we determined that the stronger the ecosystem services of the individual component types, the closer they occurred to the city centre. However, there were also certain distinct phenomena: the ecosystem services of the sports venues (SP) were lower than those of the Chinese fast-food restaurants (LZN and SXS) and express outlets (SF and STO), but they were located closer to the city centre. The target services of the sports venues were cultural services, which is more in line with the preferences of affluent people at the city centre^15^, and the sports venues hardly generated any dis-services (Fig. 2b,d). Although the ecosystem services of the DFs were higher than those of the GHs, they were located farther away from the city centre than were the GHs (Fig. 2b,d). The DFs occupied a larger share of land and had potential environmental or health impacts, leading to an extremely low ecological index. The government has standardized their functional zoning^13^. Ecosystem services are important location factors that can help us better understand the distribution of city functional components.

The locations of city functional components constantly change in response to city development. From 2012 to 2018, the functional components in cities of different sizes exhibited varying spatiotemporal dynamics: the peak position (d^*^) of the catering components (KFC, McD, LZN and SXS) in large cities moved outward across approximately 1–2 km, while the d^*^ value of the catering components in small cities remained almost unchanged (Fig. 6 and Supplementary Fig. S5). The reason for this phenomenon may be that with increasing built-up area in large cities, the location of the catering components moved outward with increasing population distribution area and the degree of agglomeration decreased^22,41^. To date, research on express outlets has focused only on metropolises with a population of approximately 10 million, such as London and Paris, and the results indicated that express outlets constantly moved towards the suburbs^23,24^. This finding is consistent with the results of our research on large Chinese cities (such as Beijing and Shanghai, with a population of approximately 20 million) (Fig. 6a,b). However, in small cities (such as Changzhou and Jiaxing with a population of approximately 5 million), the d^*^ value of the express outlets (SF and STO) moved 1.25 km towards the city centre (Fig. 6g–i). This result may be related to land rent and transport costs.

However, certain city functional components revealed the same spatiotemporal dynamic changes in the various cities of different sizes. From 2012 to 2018, the d^*^ value of the banks (ABC) in the 9 case cities remained almost unchanged, and they always occurred at the city centre (Fig. 6). This could indicate that the banking business tends to serve city centres with a high population density and high per capita income^14^. The d^*^ value of the gas stations (CNPC and Sinopec) and WTPs hardly changed (Fig. 6). The reason could be that the construction and demolition costs are high, pollution is serious, and these components occupy a large share of land. They generally require strict approval from the government, so it is difficult to change their location^42,43^. DFs are land-intensive enterprises and have increasingly moved away from the city centre (Fig. 6a–d), which is also consistent with the results of previous studies on the distribution of poultry farms^25^. The possible reason could be that with increasing urban built-up areas, an increasing area of agricultural land is developed, resulting in agricultural activities moving away from the city centre^44^. Studying the distribution and dynamic changes in city functional components along urban–rural gradients could help us comprehensively understand the evolution of urban layout. However, our findings have limitations. The study assumed that the city is monocentric, which may make the results inaccurate at a finer scale^45^. In an obviously polycentric city, it is possible to study the density distribution of city functional components on the “subcentre-edge” gradient by using the subcentre in accordance with the method described in this study to obtain a more refined regional conclusion. The analysis of component distribution in this study only focused on the gradient analysis of land prices. In the future, gradient analysis will be used in more niche dimensions (such as the population demand, transportation, policy, labour, topography, climate, and the component attributes). The method to investigate the distribution and dynamic changes of city functional components has great potential.

## Conclusions

This study focused on the distribution dynamics of 13 typical types of city functional components along the urban–rural gradient. The results showed that the density distribution curves of city functional components close to the city centre have high peak values and narrow distribution ranges. The spatiotemporal dynamics of some types of city functional components were related to the city size. In large cities, restaurants tended to move away from the city centre, whereas in small cities, the location of restaurants remained almost unchanged. The express outlets in large cities constantly left the city centre, while those in small cities tended to move towards the city centre. However, certain city functional components exhibited a consistent movement regularity among the cities of different sizes, such as banks, gas stations and WTPs, which moved little, while DFs tended to move away from the city centre. This study also introduces ecosystem services as location factors, which could be useful to explain the distribution mechanism of city functional components. This study can help us more intuitively perceive the location and movement of city functional components and better understand the development process of urban spatial structure.

## Materials and methods

### Data collection

#### Selection of the case cities and city functional components

To make the research results more universal, we set the criteria for the selection of case cities as follows. (1) Large cities: cities in which the built-up area exceeded 1000 km^2^. We chose Beijing, Shanghai, and Tianjin. Beijing is China’s capital and political centre, Shanghai is China’s largest economic centre, and Tianjin is one of China’s four municipalities directly governed by the Central Government; (2) medium cities: cities in which the built-up area varied between 400 and 1000 km^2^. We chose two provincial capital cities in central China, Wuhan and Hefei, and an economically developed coastal city, Ningbo; (3) small cities: cities in which the built-up area was smaller than 400 km^2^. Small cities need to have a complete urban form and functions. We selected three economically developed small cities Changzhou, Nantong and Jiaxing.

The selection of city functional component types should cover typical city functional components related to the coupling between humans and the city in urban systems, including production, processing, circulation, decomposition and other functions: Kentucky Fried Chicken (KFC) and McDonald's (McD), two of the most popular western fast-food restaurants in China; Lanzhou Noodles (LZN) and Shaxian Snacks (SXS), two of the most popular Chinese fast-food restaurants in China; Agricultural Bank of China (ABC), one of the four most widely distributed banks in China; swimming pool (SP), a type of indoor sports venue popular in recent years; Shunfeng (SF) and Shentong (STO) express outlets, two of the most commonly used express service components in China; China National Petroleum Corporation (CNPC) and China Petroleum and Chemical Corporation (Sinopec) gas stations, two gas station enterprises accounting for more than half of the total number of gas stations in China; WTP, a type of waste treatment component; GH, a type of primary biological production component; and DF, a type of secondary biological processing component.

#### Acquisition of city functional component data

Latitude and longitude data of the above city functional components were obtained through electronic maps and remote sensing images and verified through field investigation. AutoNavi and Baidu electronic maps are the two most widely used map suppliers in China due to their high accuracy and practicality^46^. In particular, the location of service city functional components can be accurately obtained through electronic maps. WTPs have detailed lists and location data on the government websites, and GHs can be accurately identified in Google Earth images due to their unique appearance^31^. Therefore, these three types of raw data are listed as the main sources of location data for functional components.

Latitude and longitude data of the KFC, McD, ABC, SP, LZN, SXS, SF, STO, CNPC, Sinopec and DF locations were retrieved from AutoNavi and Baidu historical electronic maps through Python 3.5 software (https://www.python.org/). The 2012 and 2015 historical electronic map data originated from the East China Normal University Humanities and Social Sciences Big Data Platform^47^, and the 2018 historical electronic map data originated from the Peking University Open Research Data Platform^48^. Based on AutoNavi and Baidu, each individual component was strictly filtered by name and type. Please refer to the Supplementary Table S3 for a summary of the detailed filtering conditions.

Accurate WTP latitude and longitude data were obtained by using the WTP name and address to query the AutoNavi map coordinate picking system [the WTP name and address were acquired from the Ministry of Ecology and Environment of the People’s Republic of China (www.mee.gov.cn), China Environment Network (www.cenews.com.cn) and Beijing Municipal Ecology and Environment Bureau (sthjj.beijing.gov.cn)]. GH latitude and longitude data were determined via a method commonly used in community ecology, which has previously been reported^31^. Briefly, ArcGIS 10.3 software was employed to generate grids covering the entire city (the size of each grid was 0.5 × 0.5 km), and these grids were then converted into the keyhole markup language (KML) format and imported into Google Earth for GH visual interpretation. The GHs were characterized as (a) bright white or bluish-white, (b) rectangular-shaped objects, (c) oriented in rows or separated by paths or bare areas. If a GH occurred in a specific grid, the centre of the grid was marked with the landmark tool to obtain the corresponding latitude and longitude data.

Land price and housing price are affected by location factors such as population, employment, transportation, and amenities and are important indicators to determine whether a city is monocentric or polycentric^49,50^. Land price was also used as a determining indicator in our study. The concentric circle model was first established by Von Thünen^51^ to study the order of agricultural land use from urban to rural areas, and it is still an important method to explore research topics along the urban–rural gradient^32,52^.

To obtain the land price distribution curve along the urban–rural gradient, all the standard land parcel information in each case city through the real-time land price query function provided by the China Land Price Information Service Platform (www.landvalue.com.cn), including land price, latitude and longitude, was obtained, and the parcel with highest land price was defined as the city centre. Concentric circles with an increasing radius of 1-km intervals were generated by adopting the city centre as the circle centre, and the average land price of all standard land parcels in each concentric ring was considered as the land price of the ring. We found that in all the case cities, the land price exhibited an obvious monotonous downward trend from the centre to the edge of the city (Supplementary Fig. S7). Therefore, we assumed a monocentric city model and used the concentric circles to define the urban–rural gradient.

To acquire density distribution curves of the city functional components along urban–rural gradients, the latitude and longitude data of the KFC, McD, ABC, SP, LZN, SXS, SF, STO, CNPC, Sinopec, GH, WTP and DF components were applied for map labelling purposes. Concentric circles with the increasing radius of 1-km intervals were generated by adopting the city centre as the circle centre, and the number of each type of component in each concentric ring was counted. Since the overall number of WTPs and DFs was smaller, the concentric circle radius was increased at 5- and 10-km intervals, respectively, and the number of WTPs or DFs in each concentric ring was determined, while the component density in each ring was calculated by dividing the number by the area of the ring.

To calculate the ecosystem services per unit area for each type of city functional component, the revenue of each component in the current year was determined. KFC and McD revenue data were retrieved from Yum China Holdings and Askci Corporation, respectively. ABC revenue data originated from the Agricultural Bank of China, Ltd., and SF and STO revenue data were acquired from SF Holding Corporation, Ltd., and STO Express Corporation, Ltd., respectively, while CNPC and Sinopec revenue data were retrieved from PetroChina Company, Ltd., and Sinopec Corporation, respectively. Moreover, LZN and SXS revenue data were obtained via field investigation. Environmental impact data of the KFC, McD, CNPC and Sinopec components originated from the Ministry of Ecology and Environment of the People’s Republic of China (www.mee.gov.cn), while LZN and SXS environmental impact data were obtained via field investigation. The costs of the KFC, McD, LZN, SXS, CNPC and Sinopec environmental impacts were converted according to the Environmental Protection Tax Law, 2018. The WTP ecosystem services were retrieved from Liu et al.^53^, and the GH ecosystem services originated from Chang et al.^54^, while the DF ecosystem services were obtained from Fan et al.^55^. The cultural services of all components were determined through field investigation.

### Data processing

To intuitively describe the density changes of city functional components along the urban–rural gradient, the density of the components in the above concentric rings were adopted as the ordinate, the distance from the city centre to the edge of the ring was adopted as the abscissa, and scatter plots were created. To compare the characteristic values of the density distribution of each type of component more clearly, a distribution model was used to fit the scatter plots^35,36^.

### Fitting of the density distribution curve of the city functional components

Through the nonlinear fitting function in OriginPro 2019 software (https://www.originlab.com/), the Gumbel model^56,57^ was considered to fit the above scatter plots to generate density distribution curves of all city functional components. The goodness-of-fit (choosing the 13 types of components in Beijing as examples) is shown in the Supplementary Fig. S2.

The component density (*P*, individual components km^−2^) at a given distance from the city centre (*d*, km) along the urban–rural gradient is calculated as follows:

1 $${P} = {P_{max}} {\cdot} {{e^{-{e}}}^{-\frac{{{d}}-{d^{*}}}{{w}} \, - \, \frac{{{d}}-{d^{*}}}{{w}} \, + \, {1}}}$$

where *P*_*max*_ (individual components km^−2^) is the peak value of the curve, d^*^ (km) is the peak position of the curve, and *w* (km) is a parameter controlling the width of the curve.

### Calculation of the niche width of the density distribution curve of the city functional components

To intuitively compare the distance spanned by the density distribution curve of the city functional components, the difference in the abscissa between a density value of 10% of the *P*_*max*_ value on the density distribution curve was adopted as the niche width *W* (km).

### Calculation of the skewness and kurtosis of the density distribution curve of the city functional components

The skewness and kurtosis are calculated according to the following equation^58^:

2 $$\text{skewness } = \frac{\frac{1}{{{n}}}{\sum }_{{{i}}= {1}}^{{n}} \,{\left({{x}}_{{i}}-{\bar{x}}\right)}^{3}}{{\left(\frac{1}{{{n}}}{\sum}_{{{i}}= {1} }^{{n}} \,{\left({{x}}_{{i}}-{\bar{x}}\right)}^{2}\right)}^{\frac{3}{{2}}}}$$

3 $$\text{kurtosis } = \frac{\frac{1}{{{n}}}{\sum }_{{{i}}= {1}}^{{n}} \,{\left({{x}}_{{i}}-{\bar{x}}\right)}^{4}}{\left(\frac{1}{n}\sum\nolimits_{i=1}^{n} \,{\left({{x}}_{{i}}-{\bar{x}}\right)}^{2}\right)^2}-3$$

where *x*_*i*_ (km) is the distance from each individual type of component to the city centre, and ‾*x* (km) is the average of the distances from all individual types of components to the city centre.

### Correlation analysis between the characteristic values of the density distribution curve

Linear and nonlinear regression analyses in Microsoft Excel 2019 were implemented to study the relationship between the characteristic values of the density distribution curve, and the regression form with the best R^2^ value was selected.

### Correlation analysis between the characteristic values of the density distribution curve and the city size

Linear and nonlinear regression analyses in Microsoft Excel 2019 were implemented to study the relationship between the characteristic values of the density distribution curve and the city size, and the regression form with the best R^2^ value was selected.

### Framework for ecosystem service assessment of the city functional components

According to the classification of the Millennium Ecosystem Assessment (MA), ecosystem services include provisioning, regulating, cultural and supporting services^59^. In this study, the ecosystem services (goods and services) provided by the city functional components (artificial ecosystems) were divided into target and accompanied services (Supplementary Fig. S6), both of which may include provisioning, regulating and cultural services.

In this study, the target services of the KFC, McD, LZN, SXS, CNPC, Sinopec, GH, and DF components were provisioning services, the target services of the ABC, SF, STO, and WTP components were regulating services, and the target services of component SP were cultural services. According to the guidance of Liu et al.^53^, the above regulating and cultural services were divided into positive and negative services (dis-services).

The net service (*NES*, USD m^−2^ yr^−1^) is the sum of the positive services (target services + positive regulating services + positive cultural services) and dis-services (negative regulating services + negative cultural services):

4 $${NES} = \sum_{{i} = 1}^{n}{ES}_{i}$$

where *ES*_*i*_ (USD m^−2^ yr^−1^) is the value of a given type of ecosystem service involved in this study, and *n* is the number of ecosystem service types involved in this study.

The ecological index (*γ*) is calculated as follows:

5 $${\gamma } = {TGS}/ |EDS|$$

where *TGS* (USD m^−2^ yr^−1^) denotes the target services of the city functional components, and *EDS* (USD m^−2^ yr^−1^) denotes the dis-services of the city functional components.

### Calculation of the ecosystem services of the city functional components

The calculation methods are provided in the supplementary materials.

## Supplementary Information

Supplementary Information.

## Data Availability

The data that support the findings of this study are available from the corresponding author upon reasonable request.
