# Supplementary material for: Studying the distribution patterns, dynamics and influencing factors of city functional components by gradient analysis
Source: Sci Rep. 2021 Sep 7;11:17802. doi: 10.1038/s41598-021-97208-4 (PMC8423789; doi:10.1038/s41598-021-97208-4)
Supplement: Supplementary file 1 — Supplementary Information. [file 41598_2021_97208_MOESM1_ESM.docx]

**Studying the distribution patterns, dynamics and influencing factors of city functional components by gradient analysis - Supplementary Materials**

Shun Liu^1^, Guofu Yang^1^, Zhaoping Wu^1^, Feng Mao^2^, Zelong Qu^1^, Ying Ge^1^ & Jie Chang^1, *^

^1^College of Life Sciences, Zhejiang University, Hangzhou, 310058, China

^2^School of Earth and Environmental Sciences, Cardiff University, Cardiff, CF10 3AT, United Kingdom

*To whom correspondence should be addressed: e-mail: jchang@zju.edu.cn

**Supplementary methods**

*Calculation of the ecosystem services of the city functional components*

***KFC, McD, LZN, and SXS***

**Target services** The target services of a fast-food restaurant are provisioning services (*ES_p_*, USD m^-2^ yr^-1^), i.e., to provide food to consumers. They were calculated as follows:

$\text{ES}_{\text{p}}\text{ = }\frac{\text{RE}_{\text{t}}}{\text{N}_{\text{t}}\text{×}\text{A}}$ (1)

where *RE_t_* (USD yr^-1^) is the total operating revenue of all restaurants of the company in one year, *N_t_* is the total number of restaurants belonging to the company, and *A* (m^2^) is the average area of the restaurants.

**Accompanied services** A fast-food restaurant provides consumers with entertainment activities, i.e., positive cultural services (*ES_c_*, USD m^-2^ yr^-1^) (calculated with the surrogate market method ^1^), such as surfing the internet, chatting, reading books, and playing games. In addition, a fast-food restaurant emits exhaust gas, wastewater, and solid waste and generates noise, which are negative regulating services (*ES_r_*, USD m^-2^ yr^-1^). They were calculated as follows:

$\text{ES}_{\text{c}}\text{ = }\frac{\text{F}\text{×}\text{T}_{\text{l}}\text{×}\text{PR}_{\text{l}}}{\text{A}}$ (2)

where *F* (person yr^-1^) is the volume of customers, *T_l_* (h person^-1^) is the leisure time per person, and *PR_l_* (USD h^-1^) is a conversion factor of the leisure time value. Here, the cost generated by the entertainment provided by an internet cafe replaces the value generated by the leisure time in a fast-food restaurant, and *A* (m^2^) is the average area of the restaurants.

$\text{ES}_{\text{r}}\text{ = }\frac{\text{EM}_{\text{wg}}\text{×}\text{PR}_{\text{wg}}\text{+}\text{EM}_{\text{ww}}\text{×}\text{PR}_{\text{ww}}\text{+EM}_{\text{sw}}\text{×}\text{PR}_{\text{sw}}\text{+}\text{ES}_{\text{no}}}{\text{A}}$ (3)

where *EM_wg_* (kg yr^-1^) is the amount of exhaust gas emissions, *EM_ww_* (kg yr^-1^) is the amount of wastewater discharge, *EM_sw_* (kg yr^-1^) is the amount of solid waste generation, and *PR_wg_* (USD kg^-1^) is the charge standard of a unit mass of exhaust gas. Moreover, *PR_ww_* (USD kg^-1^) is the charge standard of a unit mass of wastewater, *PR_sw_* (USD kg^-1^) is the charge standard of a unit mass of solid waste, *ES_no_* (USD yr^-1^) is the cost of soundproof wall construction to reduce noise, and *A* (m^2^) is the average area of the restaurants.

***ABC***

**Target services** The target services of a bank are regulating services (*ES_r_*, USD m^-2^ yr^-1^), i.e., to raise and distribute funds. They were calculated as follows:

$\text{ES}_{\text{r}}\text{ = }\frac{\text{RE}_{\text{t}}}{\text{N}_{\text{t}}\text{×}\text{A}}$ (4)

where *RE_t_* (USD yr^-1^) is the total operating revenue of all business locations of the company in one year, *N_t_* is the total number of banks belonging to the company, and *A* (m^2^) is the average area of the banks.

**Accompanied services** Customers experience much waiting time before conducting business in a bank, i.e., negative cultural services (*ES_c_*, USD m^-2^ yr^-1^) (calculated with the surrogate market method ^1^). They were calculated as follows:

$\text{ES}_{\text{c}}\text{ = }\frac{\text{F}\text{×}\text{T}_{\text{w}}\text{×}\text{PR}_{\text{w}}}{\text{A}}$ (5)

where *F* (person yr^-1^) is the volume of customers, *T_w_* (h person^-1^) is the waiting time per person, and *PR_w_* (USD h^-1^) is a conversion factor of the value loss caused by waiting. Here, the salary covering the same working time replaces the value of the waiting time, and *A* (m^2^) is the average area of the banks.

***SP***

**Target services** The target services of a swimming pool (SP) are cultural services (*ES_c_*, USD m^-2^ yr^-1^), i.e., to provide people with fitness and entertainment opportunities. They were calculated as follows:

$\text{ES}_{\text{c}}\text{ = }\frac{\text{RE}}{\text{A}}$ (6)

where *RE* (USD yr^-1^) is the operating revenue of a venue in one year, and *A* (m^2^) is the area of a SP.

**Accompanied services** Water evaporation in SPs adjusts their microclimate, i.e., positive regulating services (*ES_r_*, USD m^-2^ yr^-1^) (calculated with the surrogate market method ^1^). They were calculated as follows:

$\text{ES}_{\text{r}}\text{ = }\frac{\text{Q}_{\text{w}}\text{×}\text{PR}_{\text{e}}}{\text{A}}$ (7)

where *Q_w_* (kJ yr^-1^) is the heat consumption of water evaporation and *PR_e_* (USD kJ^-1^) is the charge standard of electricity. Here, the cost of the equivalent energy consumption of air conditioning replaces the heat consumption value of water evaporation, and *A* (m^2^) is the average area of the SPs.

***SF and STO***

**Target services** The target services of an express outlet are regulating services (*ES_r_*, USD m^-2^ yr^-1^), i.e., to distribute goods. They were calculated as follows:

$\text{ES}_{\text{r}}\text{ = }\frac{\text{RE}_{\text{t}}}{\text{N}_{\text{t}}\text{×}\text{A}}$ (8)

where *RE_t_* (USD yr^-1^) is the total operating revenue of all outlets of the company in one year, *N_t_* is the total number of outlets belonging to the company, and *A* (m^2^) is the average area of the express outlets.

**Accompanied services** Consumers experience satisfaction upon package receipt, i.e., positive cultural services (*ES_c_*, USD m^-2^ yr^-1^) (calculated with the surrogate market method ^1^). In addition, an express outlet emits exhaust gas and solid waste (not measured in this article) and generates noise, i.e., negative regulating services (*ES_r_*, USD m^-2^ yr^-1^). They were calculated as follows:

$\text{ES}_{\text{c}}\text{ = }\frac{\text{N}_{\text{d}}\text{×}\text{PR}_{\text{u}}}{\text{A}}$ (9)

where *N_d_* (pieces yr^-1^) is the delivery volume of an express outlet in one year and *PR_u_* (USD pieces^-1^) is the value of the satisfaction generated in consumers upon package receipt, and *A* (m^2^) is the average area of the express outlets.

$\text{ES}_{\text{r}}\text{ = }\frac{\text{EM}_{\text{wg}}\text{×}\text{PR}_{\text{wg}}\text{+EM}_{\text{sw}}\text{×}\text{PR}_{\text{sw}}\text{+}\text{ES}_{\text{no}}}{\text{A}}$ (10)

where *EM_wg_* (kg yr^-1^) is the amount of exhaust gas emissions, *EM_sw_* (kg yr^-1^) is the amount of solid waste generation, *PR_wg_* (USD kg^-1^) is the charge standard of a unit mass of exhaust gas, *PR_sw_* (USD kg^-1^) is the charge standard of a unit mass of solid waste, *ES_no_* (USD yr^-1^) is the cost of soundproof wall construction to reduce noise, and *A* (m^2^) is the average area of the express outlets.

***CNPC and Sinopec***

**Target Services** The target services of a gas station are provisioning services (*ES_p_*, USD m^-2^ yr^-1^), i.e., to provide vehicle refuelling opportunities to consumers, which were calculated as follows:

$\text{ES}_{\text{p}}\text{ = }\frac{\text{RE}_{\text{t}}}{\text{N}_{\text{t}}\text{×}\text{A}}$ (11)

where *RE_t_* (USD yr^-1^) is the total operating revenue of all gas stations of the company in one year, *N_t_* is the total number of gas stations belonging to the company, and *A* (m^2^) is the average area of the gas stations.

**Accompanied services** A gas station provides consumers with car cleaning services, i.e., positive cultural services (not measured in this article), but consumers experience some waiting time before refuelling, i.e., negative cultural services (calculated with the surrogate market method ^1^). Additionally, a gas station contains a certain greening area, which achieves carbon sequestration, air filtering, noise reduction and microclimate regulation, i.e., positive regulating services. However, a gas station also emits exhaust gas and wastewater and generates noise, i.e., negative regulating services. The cultural services (*ES_c_*, USD m^-2^ yr^-1^) and regulating services (*ES_r_*, USD m^-2^ yr^-1^) were calculated as follows:

$\text{ES}_{\text{c}}\text{ = }\frac{\text{F}\text{×}\text{T}_{\text{w}}\text{×}\text{PR}_{\text{w}}}{\text{A}}$ (12)

where *F* (person yr^-1^) is the volume of customers, *T_w_* (h person^-1^) is the waiting time per person, and *PR_w_* (USD h^-1^) is a conversion factor of the value loss caused by waiting. Here, the salary covering the same working time replaces the value of the waiting time, and *A* (m^2^) is the average area of the gas stations.

$\text{ES}_{\text{r}}\text{ =}\text{ }\text{ES}_{\text{gs}}\text{+ }\frac{\text{EM}_{\text{wg}}\text{×}\text{PR}_{\text{wg}}\text{+}\text{EM}_{\text{ww}}\text{×}\text{PR}_{\text{ww}}\text{+}\text{ES}_{\text{no}}}{\text{A}}$ (13)

where *ES_gs_* (USD m^-2^ yr^-1^) denotes the positive regulating services generated by the green spaces (for the calculation method, please refer to Chang et al. ^2^), *EM_wg_* (kg yr^-1^) is the amount of exhaust gas emissions, *EM_ww_* (kg yr^-1^) is the amount of wastewater discharge, and *PR_wg_* (USD kg^-1^) is the charge standard of a unit mass of exhaust gas. Moreover, *PR_ww_* (USD kg^-1^) is the charge standard of a unit mass of wastewater, *ES_no_* (USD yr^-1^) is the cost of soundproof wall construction to reduce noise, and *A* (m^2^) is the average area of the gas stations.

***WTP***

**Target services** The target services of a wastewater treatment plant (WTP) are regulating services (*ES_r_*, USD m^-2^ yr^-1^), i.e., to improve the water quality. They were calculated as follows:

$\text{ES}_{\text{r}}\text{ = }\frac{\text{PR}_{\text{wt}}\text{×}\text{Y}_{\text{wt}}}{\text{A}}$ (14)

where *PR_wt_* (USD t^-1^) is the treatment price of wastewater per unit mass, *Y_wt_* (t yr^-1^) is the amount of wastewater treated by a WTP in one year, and *A* (m^2^) is the area of a WTP.

**Accompanied services** A WTP regulates the water volume, i.e., positive regulating services. However, a WTP also emits exhaust gas, i.e., negative regulating services. For the calculation method, please refer to Liu et al. ^3^.

***GH***

**Target services** The target services of a greenhouse (GH) are provisioning services (*ES_p_*, USD ha^-1^ yr^-1^), i.e., to supply people with crops. They were calculated as follows:

$\text{ES}_{\text{p}}\text{= }\frac{\sum_{\text{sp}\text{ =}\text{1}}^{\text{n}} \text{Y}_{\text{sp}}\text{×}\text{PR}_{\text{sp}\text{ }}}{\text{A}}$ (15)

where *Y_sp_* (t yr^-1^) is the economic production of crop species *sp*, *PR_sp_* (USD t^-1^) is the average price of crop species *sp*, and *A* (ha) is the area of a GH.

**Accompanied services** A GH achieves the functions of carbon sequestration, soil conservation, soil fertility protection, and water savings, which are positive regulating services. In contrast, a GH also causes soil salinization, generates plastic waste, and produces CH_4_ and N_2_O emissions, which are negative regulating services. For the calculation method, please refer to Chang et al. ^4^.

***DF***

**Target services** The target services of a dairy farm (DF) are provisioning service (*ES_p_*, USD ha^-1^ yr^-1^), i.e., to supply milk. The milk yield (*Y_m_*, t ha^-1^ yr^-1^) was calculated as follows:

$\text{Y}_{\text{m}}\text{ = }\frac{\text{Y}_{\text{lc}}\text{×}\text{N}_{\text{lc}}}{\text{A}}$ (16)

where *Y_lc_* (t head^-1^ yr^-1^) is the average annual yield of lactating cows, *N_lc_* (head) is the number of lactating cows in a DF, and *A* (ha) is the area of the DF. The provisioning services (*ES_p_*, USD ha^-1^ yr^-1^) were calculated as follows:

$\text{ES}_{\text{p}}\text{ = }\text{Y}_{\text{m}}\text{ × }\text{PR}_{\text{m}}$ (17)

where *Y_m_* (t ha^-1^ yr^-1^) is the milk yield per unit farm area, and *PR_m_* (USD t^-1^) is the price of milk.

**Accompanied services** A DF emits NH_3_ and greenhouse gases, which are harmful to humans and ecosystems, i.e., negative regulating services. For the calculation method, please refer to Fan et al. ^5^.


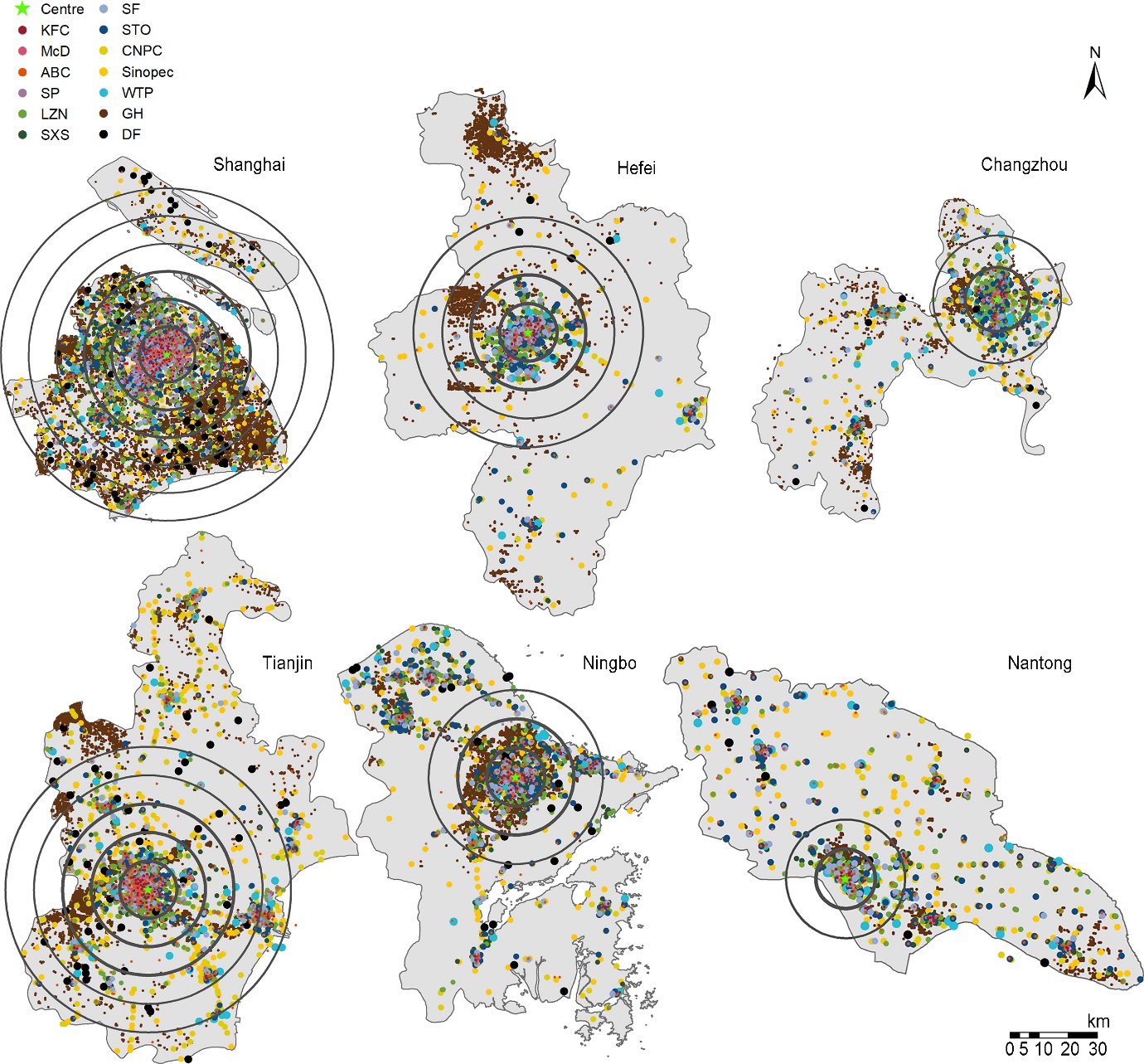


**Figure S1. Spatial point distribution of the various city functional components.** The concentric rings are drawn from the city centre to the city edge, which are centred on the city centre, and the radius of the concentric rings is increased at 10-km intervals. The maps were created using ArcGIS 10.3 software (http://www.esri.com/arcgis/).


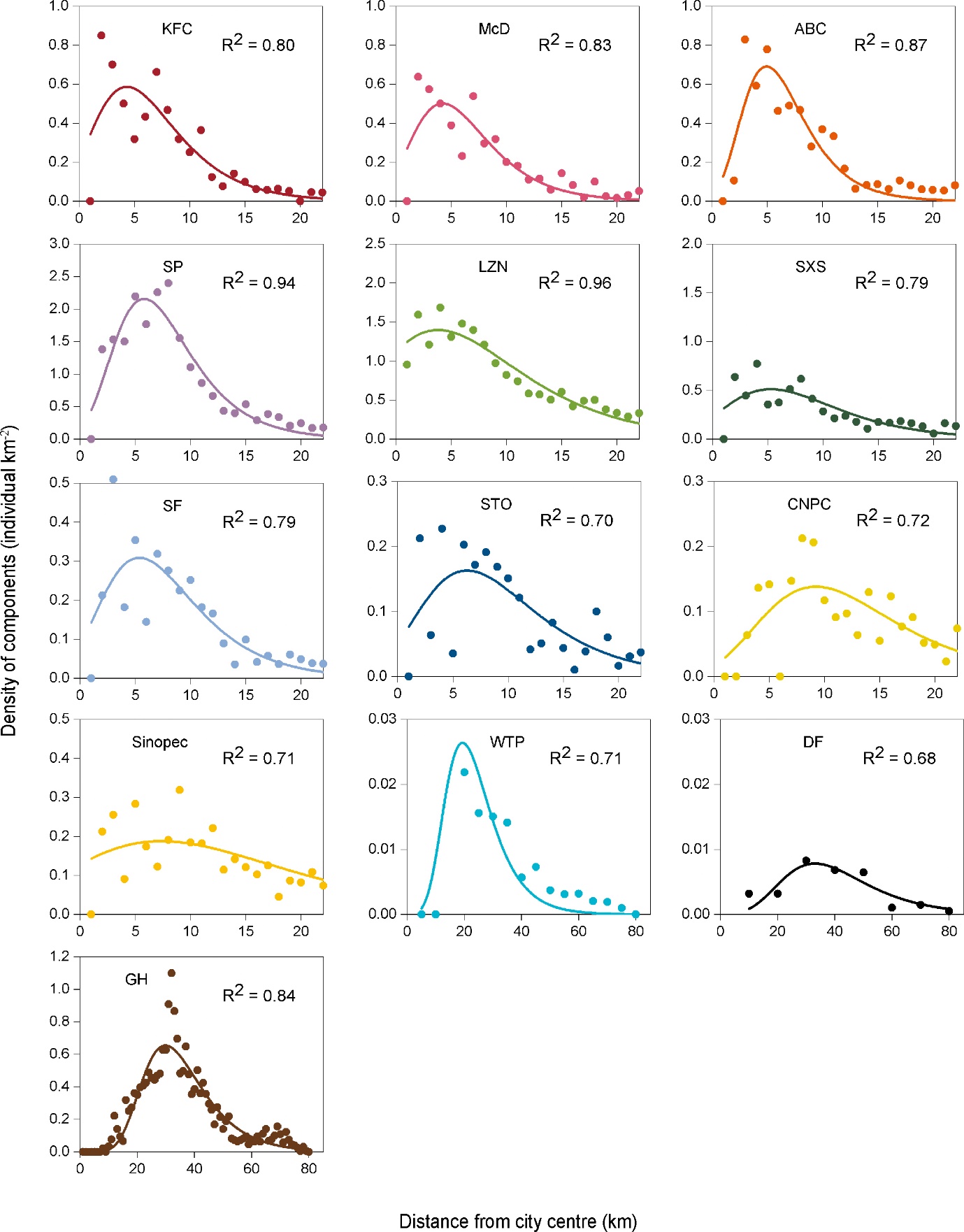


**Figure S2. Goodness-of-fit of the Gumbel model. Thirteen types of components in Beijing in 2018 are shown as an example.**


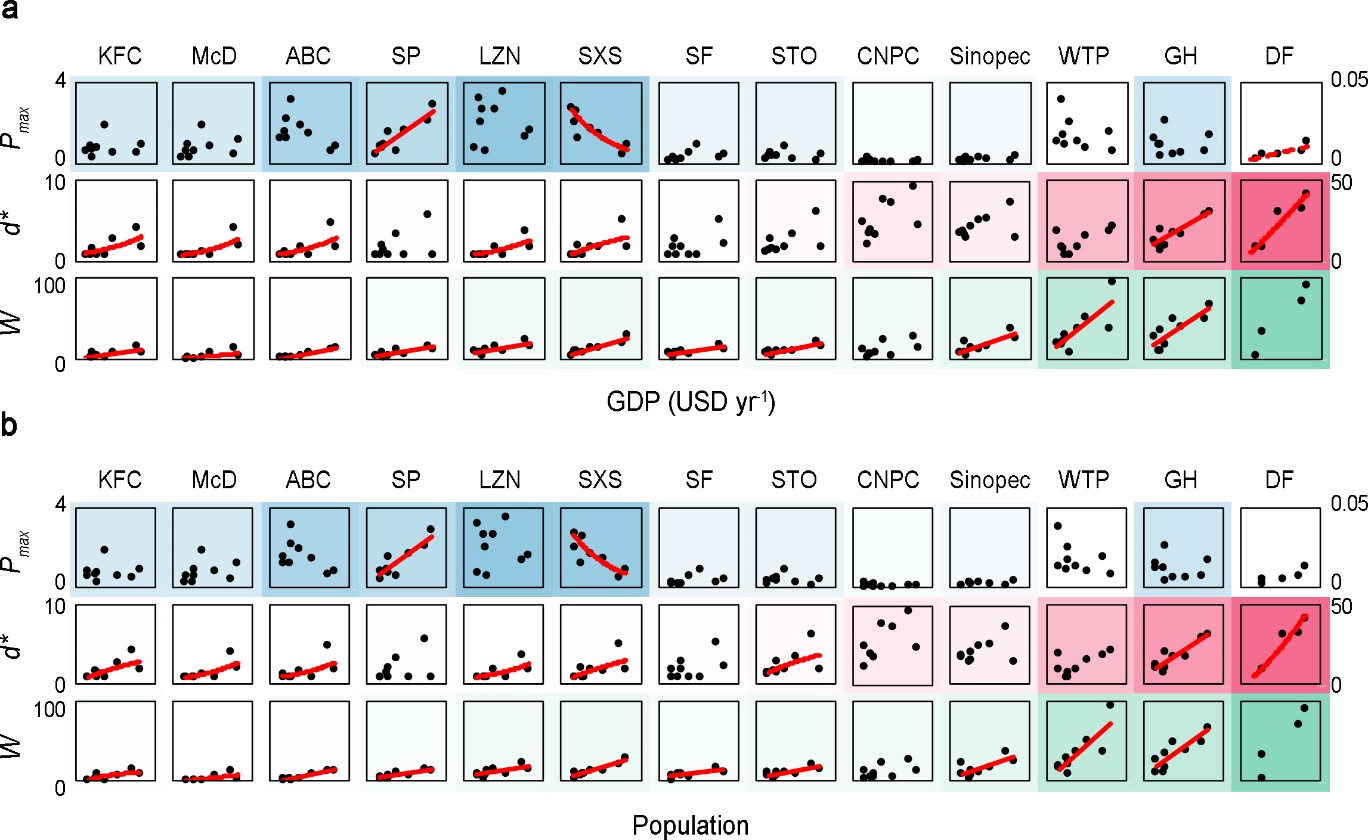


**Figure S3. Relationship between the characteristic values (*P_max_*, *d^*^*, *W*) of the density distribution curves of the city functional components and the city size (GDP or population).** (**a**) Relationship between the characteristic values of the density distribution curves of functional components and GDP. (**b**) Relationship between the characteristic values of the density distribution curves of functional components and the population. The WTP and DF components use secondary coordinates. The red line indicates the results of correlation analysis of the scatter points in the figure, all p-values < 0.05. The depth of the background colour indicates the mean value of the ordinate of the scatter points in each small figure. The abbreviations of the component names are the same as those in Figure 1 of the main text.


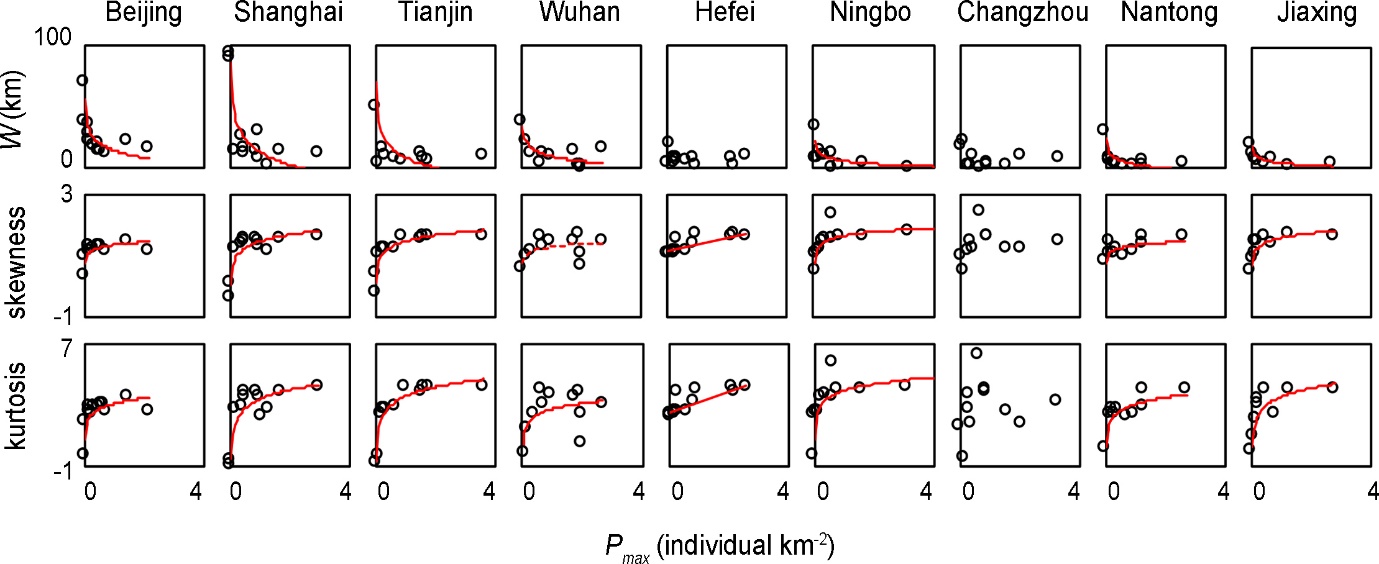


**Figure S4. Relationships between *P_max_* and the other characteristic values (*W*, skewness, and kurtosis) of the density distribution curves of the city functional components.** The red line indicates the results of correlation analysis of the scatter points in the figure, all p-values < 0.05.


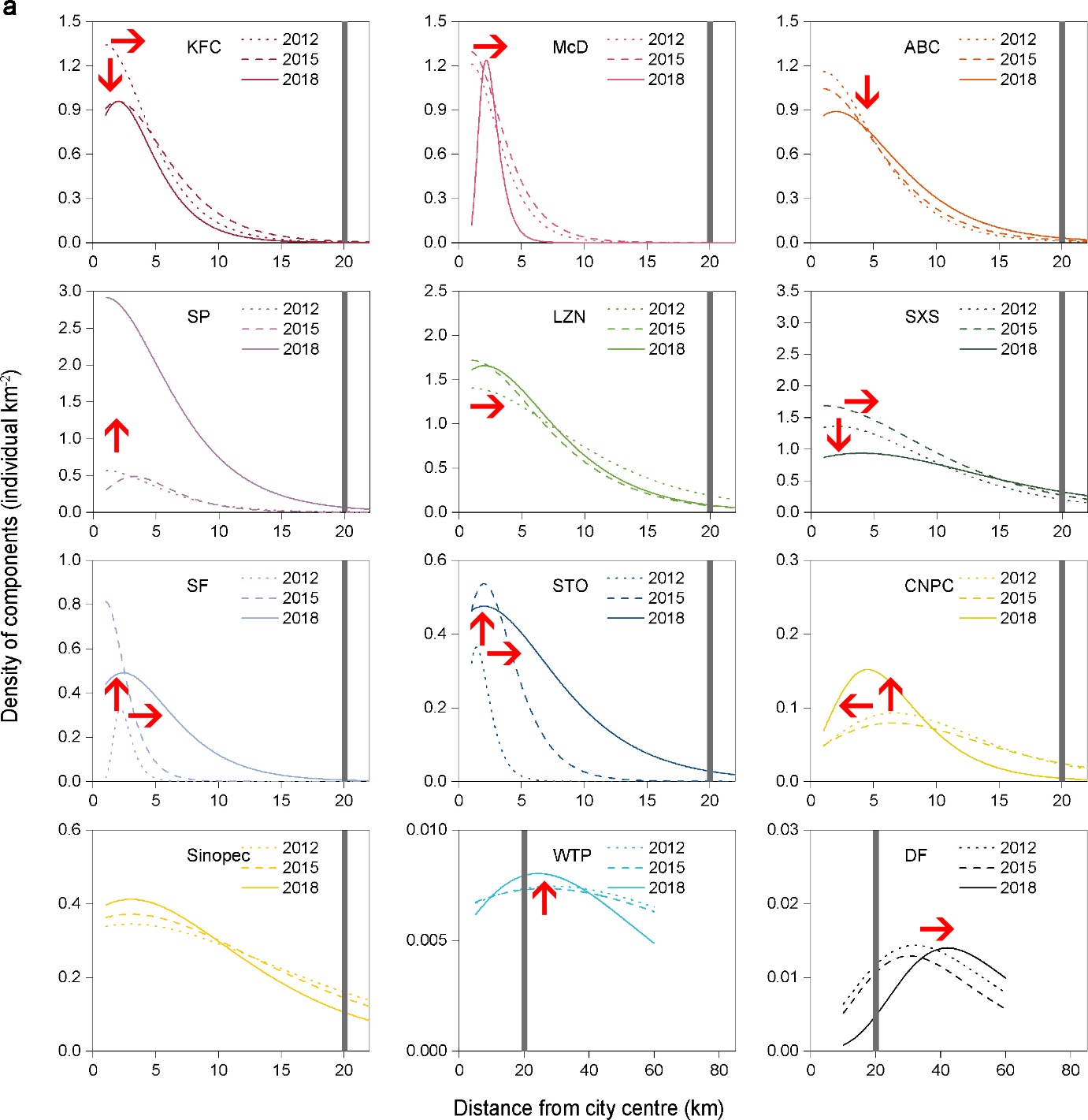


**Figure S5. Dynamic changes in the density distribution curves of the city functional components along the urban-rural gradient over time.** The arrow indicates the change trend of the density distribution curve of the city functional components. The grey band indicates the boundary of the urban built-up area. The abbreviations of the component names are the same as those in Figure 1 of the main text. (**a**) Shanghai; (**b**) Tianjin; (**c**) Wuhan; (**d**) Hefei; (**e**) Ningbo; (**f**) Changzhou; (**g**) Nantong; (**h**) Jiaxing.


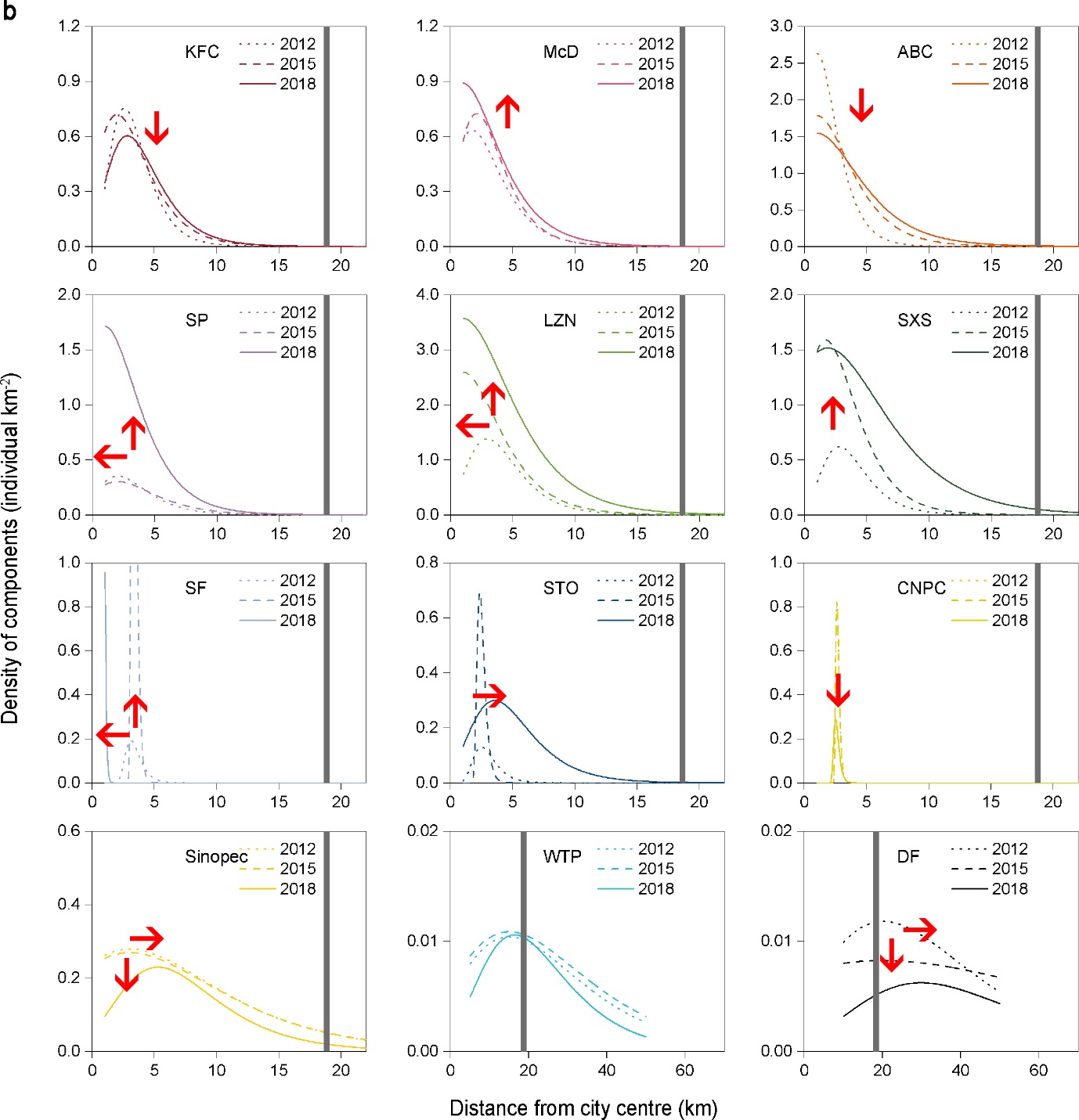


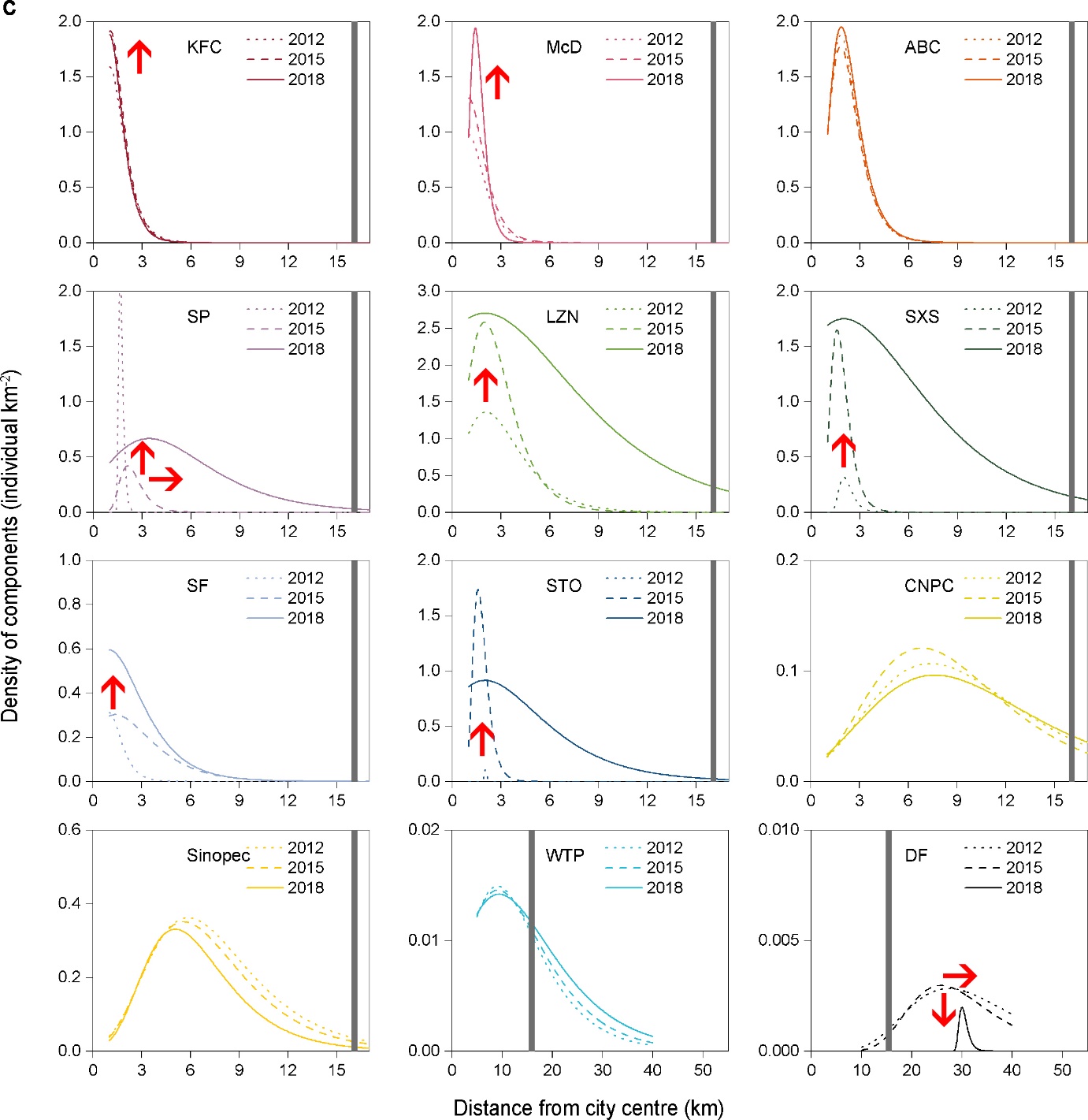


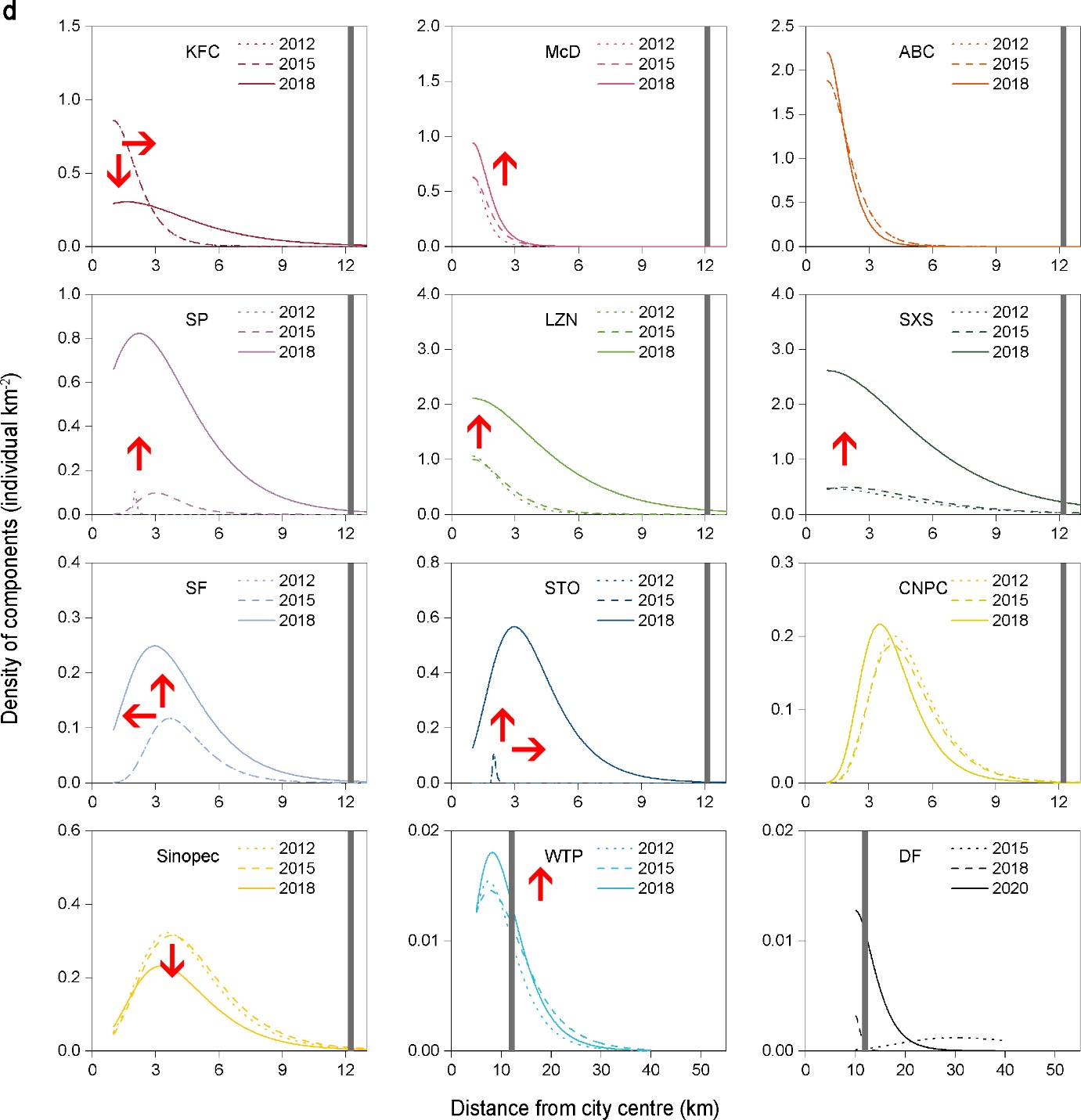


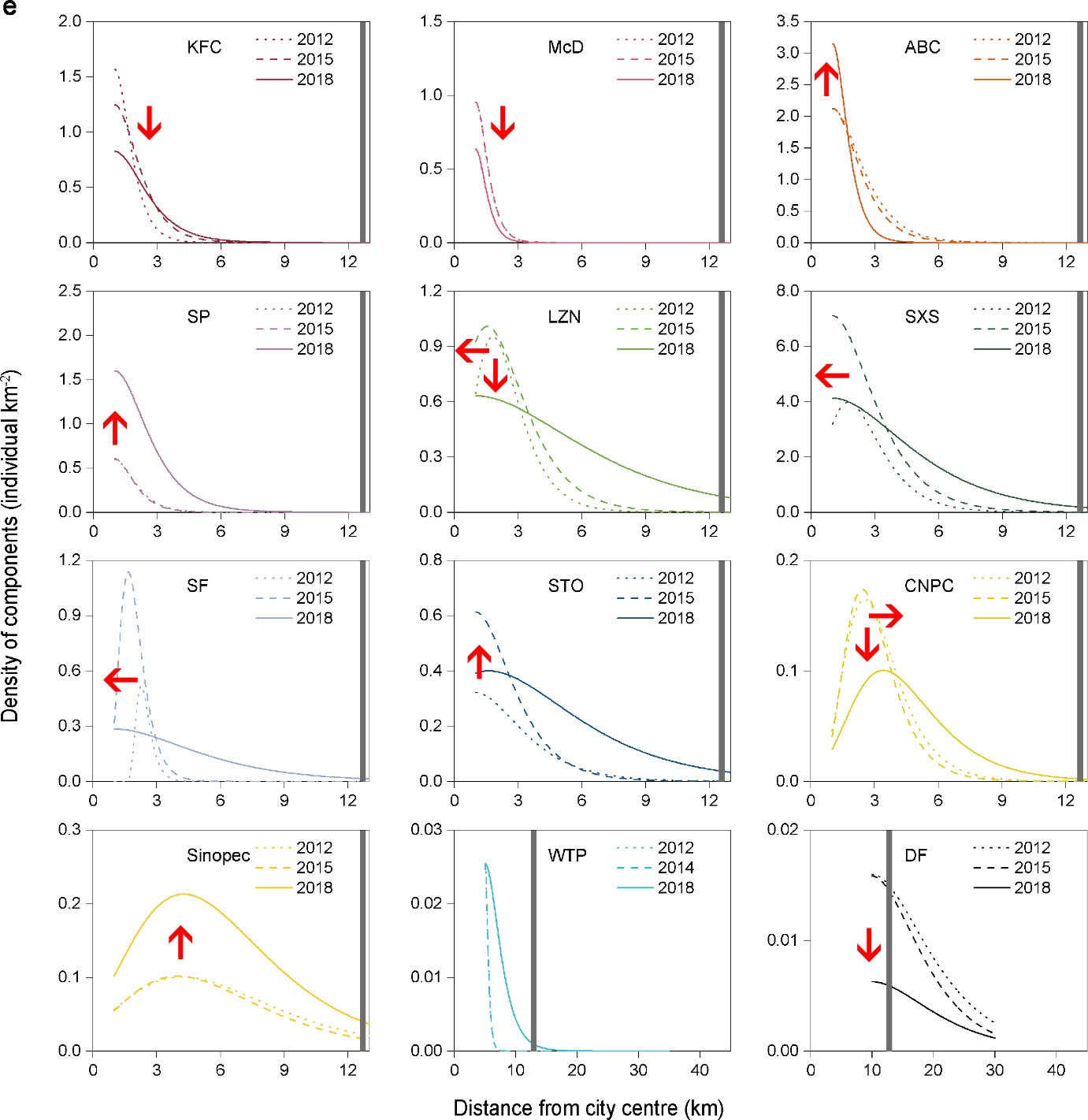


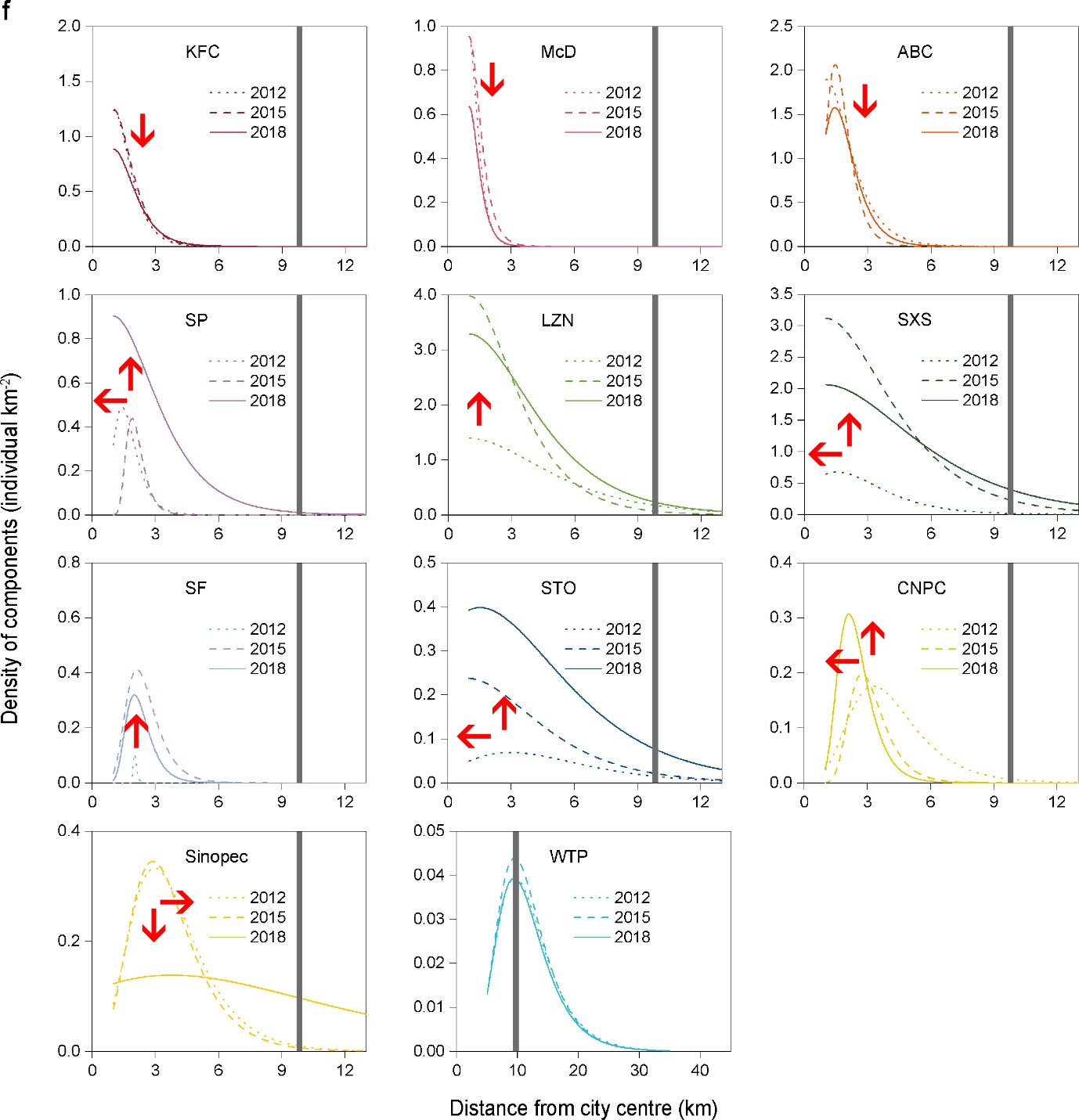


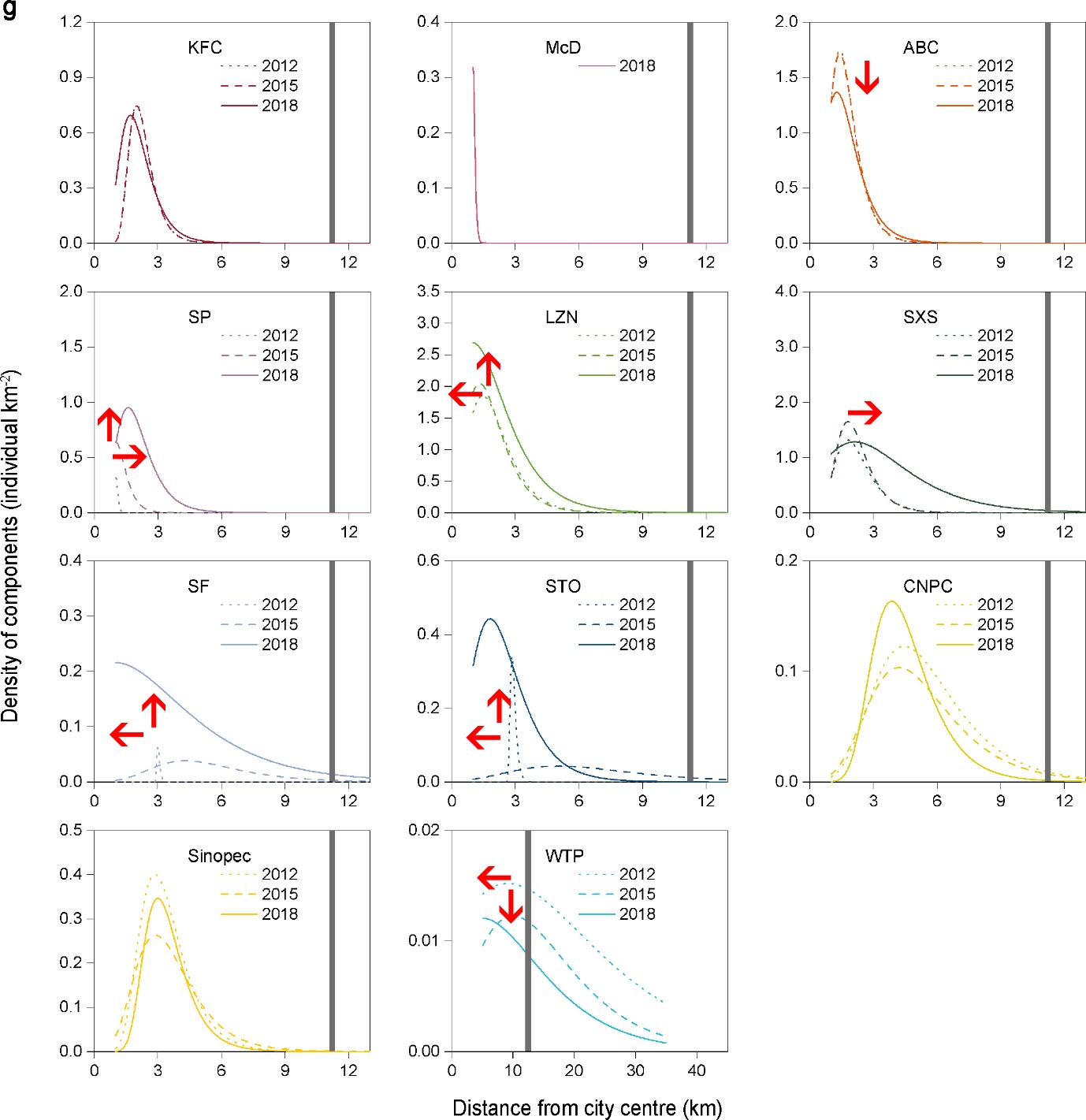


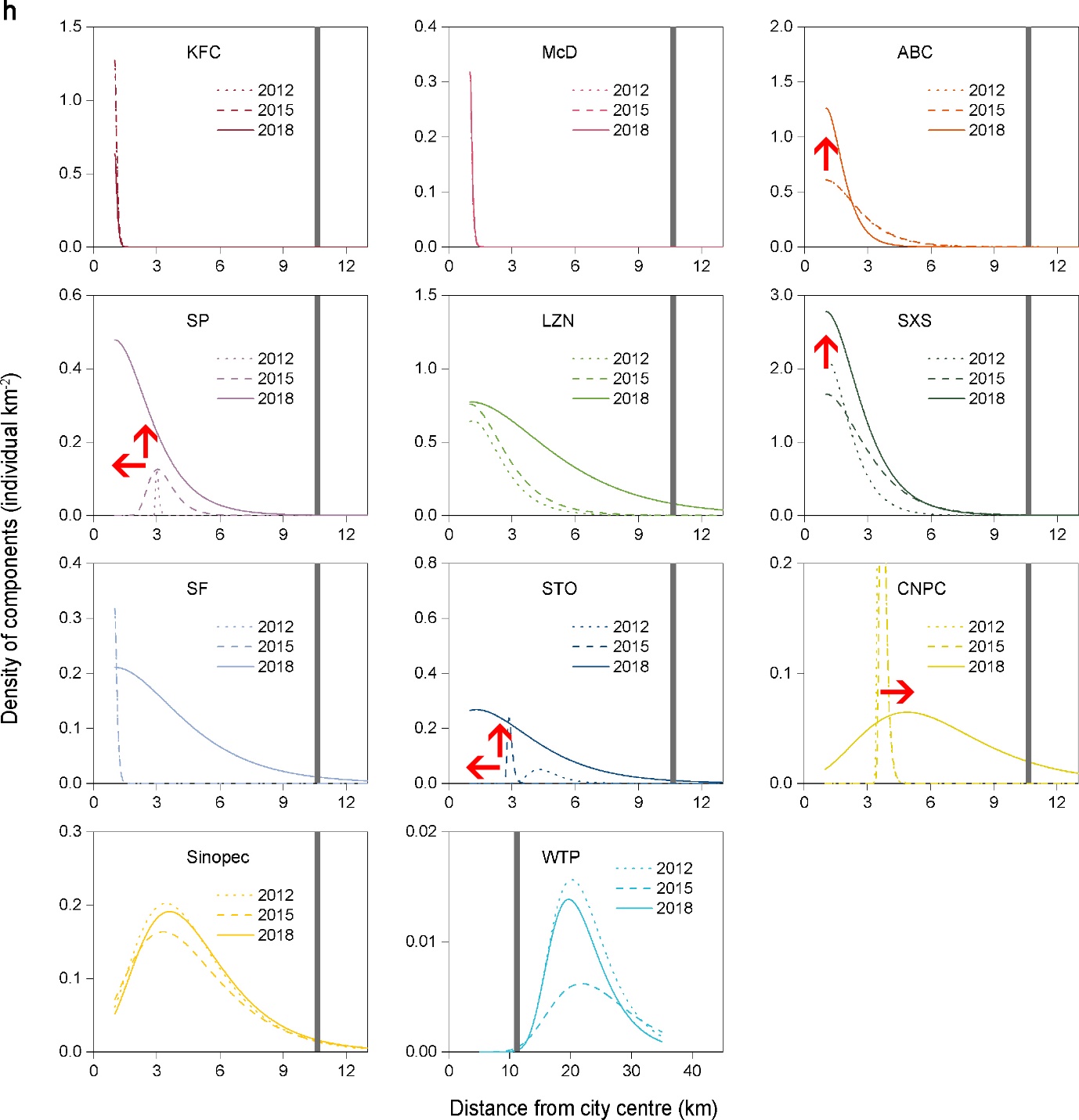


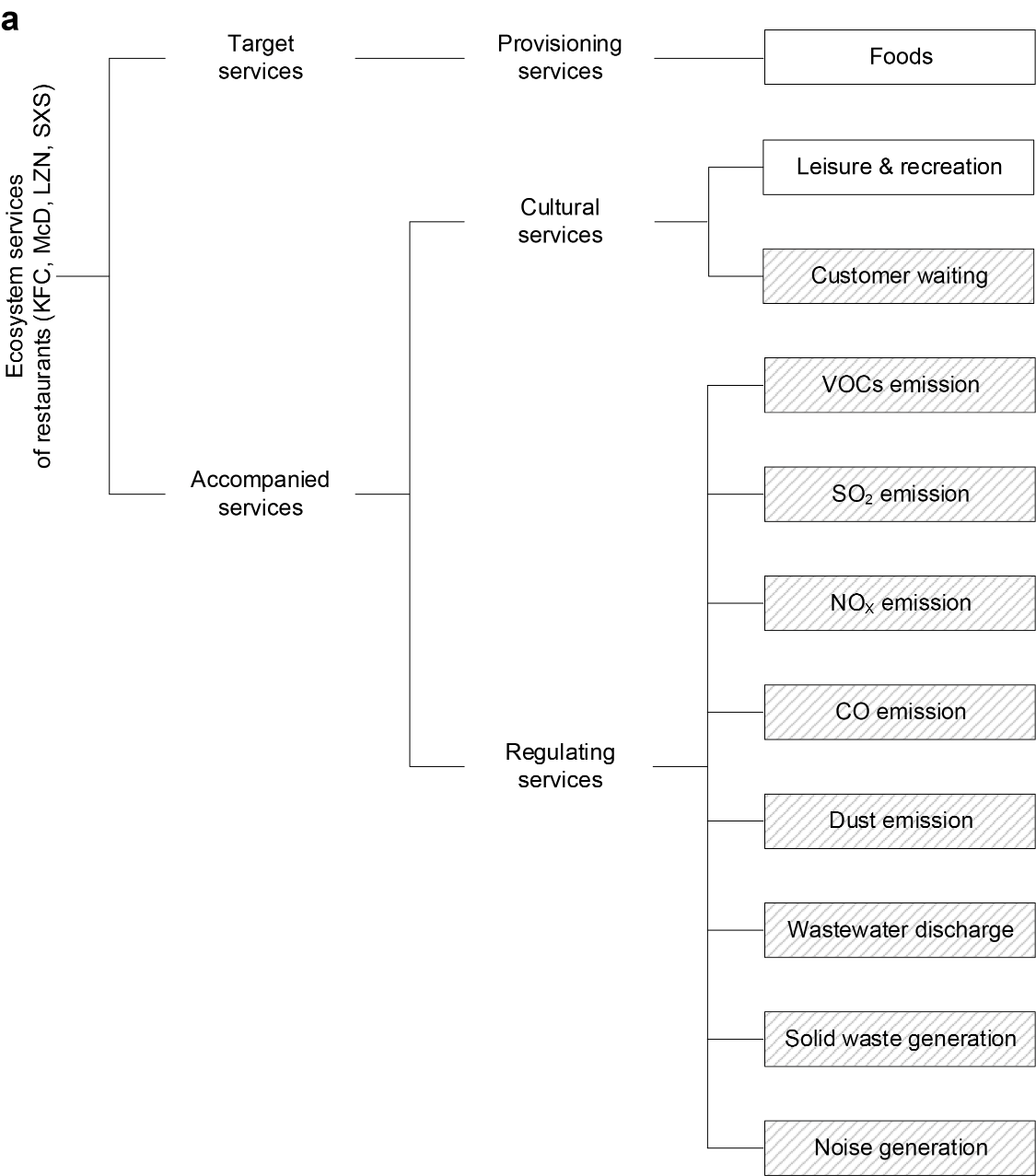


**Figure S6. Framework for ecosystem service assessments of the 13 types of city functional components in this study.** The shadow-filled text box denotes the dis-services. (**a**) Fast-food restaurants (KFC, McD, LZN, and SXS); (**b**) bank (ABC); (**c**) swimming pool (SP); (**d**) express outlets (SF and STO); (**e**) gas stations (CNPC and Sinopec); (**f**) wastewater treatment plant (WTP); (**g**) dairy farm (DF); (**h**) greenhouse (GH). The abbreviations of the component names are the same as those in Figure 1 of the main text.


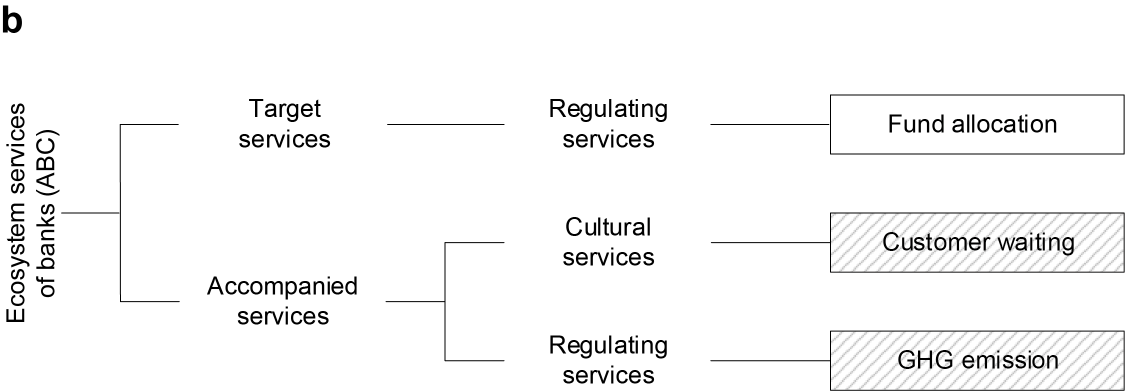


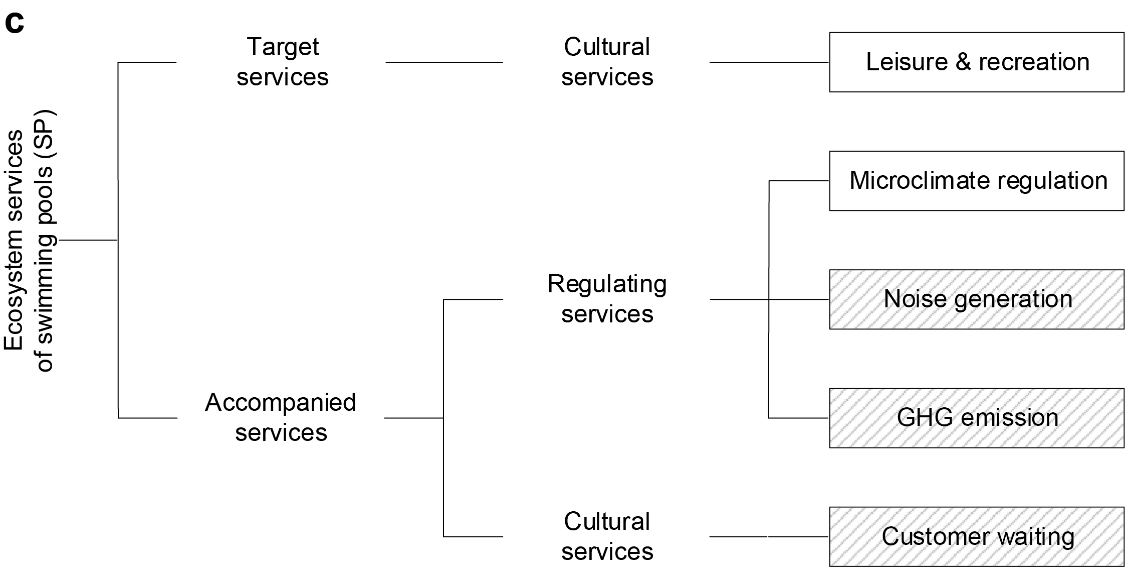


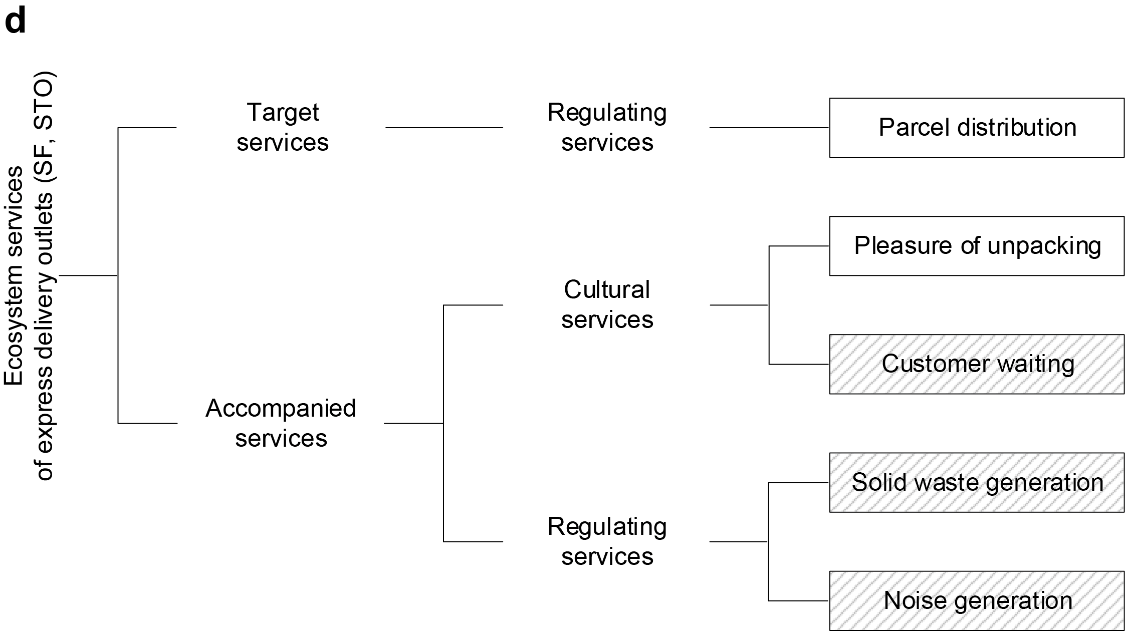


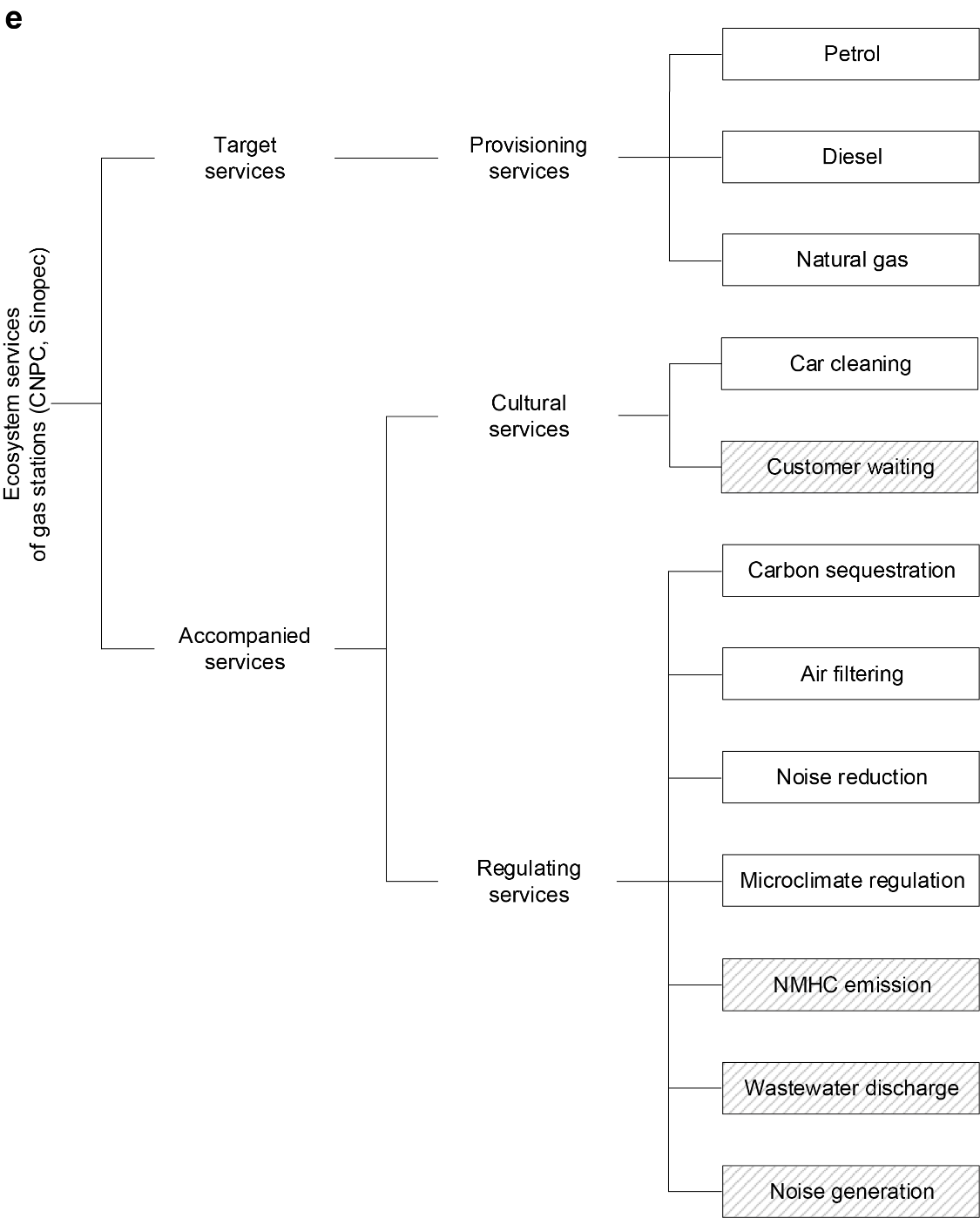


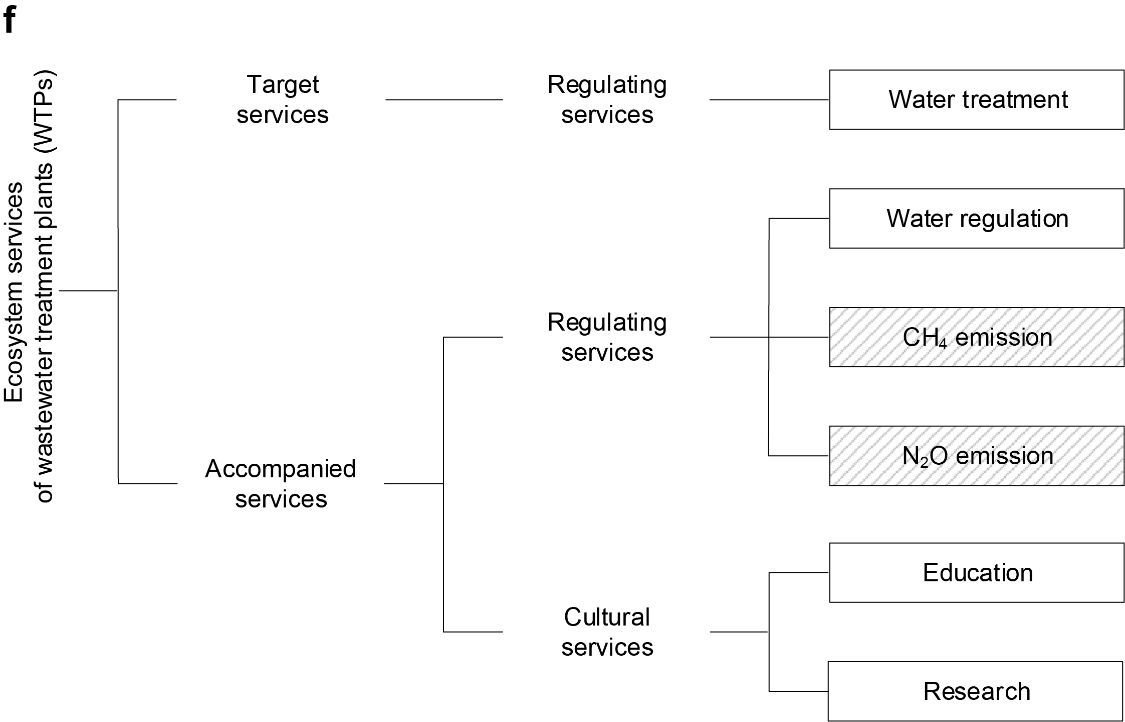


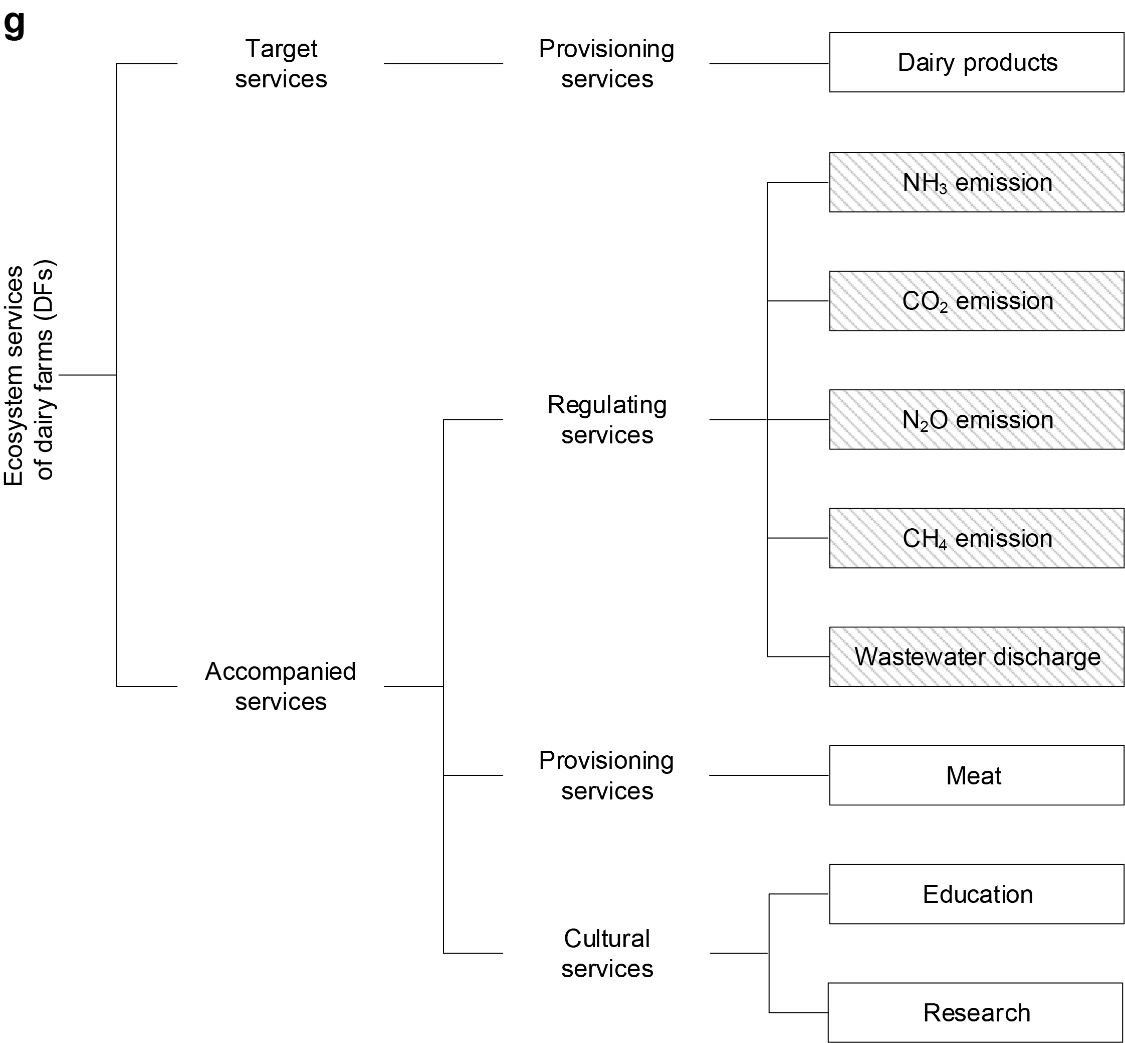


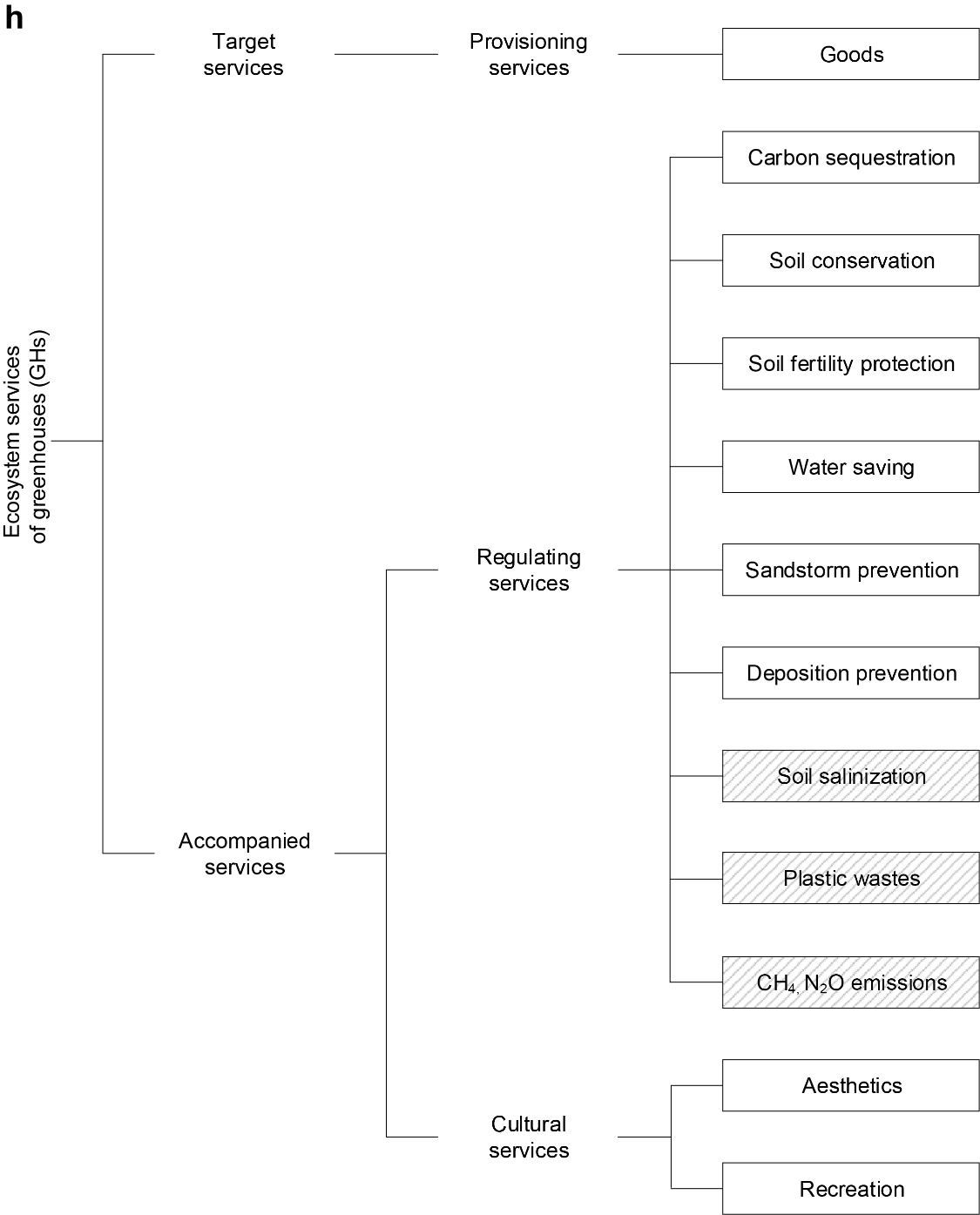


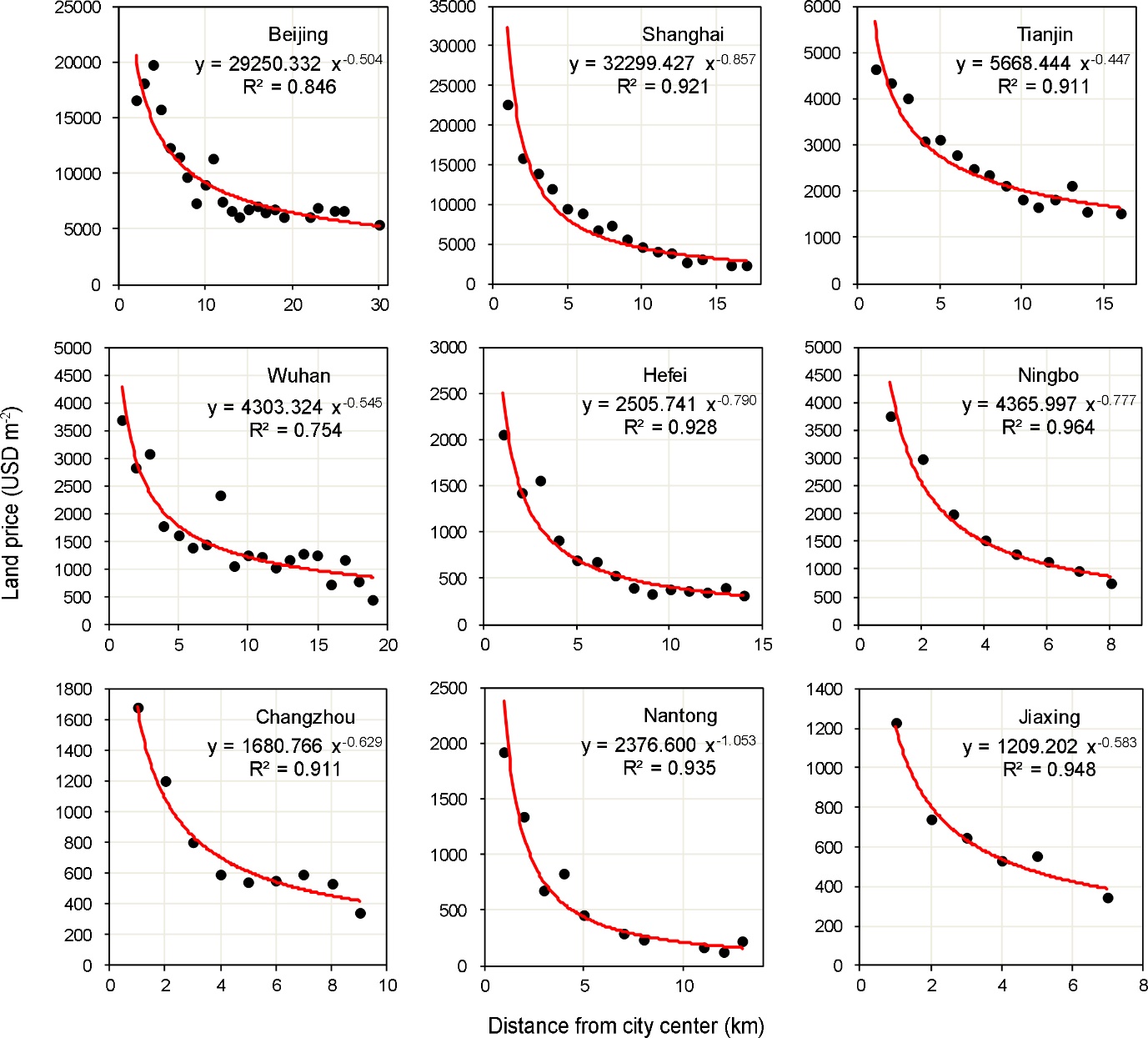


**Figure S7. Spatial distribution curves of the land price along the urban-rural gradient in the 9 case cities in China.** The data points are fitted with the power function, and the red fitting lines are all extremely significant. The cities are Beijing, Shanghai, Tianjin, Wuhan, Hefei, Ningbo, Changzhou, Nantong, Jiaxing.

**Table S1. Distribution curve characteristics of the various types of components in the 9 cities (2018).**

| Type | Width (km) | Skewness | Kurtosis |
| --- | --- | --- | --- |
| KFC | 6.60 [2.04, 15.62] | 1.47 [1.03, 1.73] | 3.32 [2.13, 3.91] |
| McD | 4.54 [1.19, 14.47] | 1.63 [0.68, 2.46] | 3.66 [0.41, 6.14] |
| ABC | 5.80 [1.72, 14.58] | 1.53 [1.14, 1.83] | 3.42 [2.35, 4.08] |
| SP | 8.15 [3.00, 16.16] | 1.48 [1.17, 1.65] | 3.42 [2.30, 4.12] |
| LZN | 12.10 [4.17, 23.42] | 1.56 [1.40, 1.65] | 3.47 [2.26, 4.09] |
| SXS | 13.52 [4.08, 30.52] | 1.50 [1.29, 1.65] | 3.20 [1.65, 4.10] |
| SF | 9.15 [2.61, 18.19] | 1.46 [1.22, 1.63] | 3.19 [2.55, 3.99] |
| STO | 11.22 [4.43, 22.19] | 1.39 [1.16, 1.58] | 2.94 [1.70, 3.79] |
| CNPC | 11.93 [3.34, 27.81] | 1.14 [0.92, 1.28] | 2.21 [0.92, 2.73] |
| Sinopec | 16.89 [4.19, 37.67] | 1.16 [0.55, 1.46] | 2.11 [-0.60, 2.77] |
| WTP | 35.78 [8.98, 94.93] | 0.78 [0.17, 1.14] | 0.69 [-0.96, 2.40] |
| GH | 34.65 [11.01, 68.15] | 0.49 [-0.21, 0.98] | 0.08 [-0.74, 1.36] |
| DF | 61.39 [3.89, 106.74] | 0.31 [-0.31, 1.10] | 0.02 [-0.93, 2.38] |

* The types of components and abbreviations are the same as those in Figure 1 of the main text.

† The values in brackets denote the range of the characteristic values between the different cities.

**Table S2. Distribution curve characteristics of the various types of components in Beijing over time (from 2012 to 2018).**

| Type | Width (km) | Skewness | Kurtosis |
| --- | --- | --- | --- |
| KFC | 15.15 [14.19, 15.62] | 1.38 [1.38, 1.34] | 3.04 [3.04, 2.92] |
| McD | 12.43 [9.03, 14.47] | 1.28 [1.23, 1.32] | 2.74 [2.58, 2.84] |
| ABC | 13.75 [13.17, 13.08] | 1.20 [1.20, 1.18] | 2.52 [2.51, 2.48] |
| SP | 14.66 [12.28, 16.16] | 1.29 [1.34, 1.20] | 2.77 [2.91, 2.51] |
| LZN | 24.23 [26.79, 23.42] | 1.49 [1.56, 1.51] | 3.40 [3.56, 3.49] |
| SXS | 19.15 [16.83, 20.71] | 1.23 [1.16, 1.37] | 2.62 [2.42, 2.99] |
| SF | 13.57 [11.95, 18.19] | 1.17 [1.17, 1.30] | 2.44 [2.46, 2.77] |
| STO | 14.79 [11.27, 22.19] | 1.20 [1.15, 1.31] | 2.53 [2.41, 2.80] |
| CNPC | 28.97 [30.68, 27.81] | 1.22 [1.22, 1.21] | 2.48 [2.47, 2.49] |
| Sinopec | 37.41 [38.07, 37.67] | 1.29 [1.28, 1.34] | 2.25 [2.27, 2.41] |
| WTP | 41.37 [49.04, 38.47] | 1.02 [0.93, 1.06] | 1.58 [1.09, 1.78] |
| DF | 53.12 [37.15, 71.59] | 0.75 [1.05, 0.41] | 0.70 [1.73, -0.36] |

* The types of components and abbreviations are the same as those in Figure 1 of the main text.

† The values in brackets denote the range of the characteristic values over time.

**Table S3. Filter conditions to obtain the required individual component types from the point-of-interest (POI) data of AutoNavi or Baidu electronic maps.**

| Type | Filter conditions |
| --- | --- |
| KFC | Field "Name" includes "Kentucky Fried Chicken \| KFC \| kfc" but not "restroom \| toilet \| park \| entrance \| exit".  Field "Type" includes "catering \| restaurant \| delicious food \| fast food". |
| McD | Field "Name" includes "McDonald's \| McDonald" but not "restroom \| toilet \| park \| entrance \| exit".  Field "Type" includes "catering \| restaurant \| delicious food \| fast food". |
| ABC | Field "Name" includes "agricultural bank" but not "off-premise \| twenty-four \| 24 \| ATM \| atm \| self-help \| automatic \| park \| entrance \| exit".  Field "Type" includes "finance \| bank" but not "ATM \| atm \| automatic teller machine". |
| SP | Field "Name" includes "swimming" but not "children \| baby \| parent-child \| restroom \| toilet \| park \| entrance \| exit".  Field "Type" includes "science, education and culture \| life services \| sports \| fitness \| leisure" but not "shower \| ticket". |
| LZN | Field "Name" includes "Lanzhou & noodles" but not "batching \| distribution".  Field "Type" includes "catering". |
| SXS | Field "Name" includes "Shaxian" but not "batching \| distribution".  Field "Type" includes "catering". |
| SF | Field "Name" includes "Shunfeng" and "express \| logistics \| deliver \| agent \| collection \| send \| take \| parcel \| package".  Field "Type" includes "life services \| company \| express \| logistics \| transport". |
| STO | Field "Name" includes "Shentong" and "express \| logistics \| deliver \| agent \| collection \| send \| take \| parcel \| package".  Field "Type" includes "life services \| company \| express \| logistics \| transport". |
| CNPC | Field "Name" includes "PetroChina" but not "card-selling \| recharge \| sales service centre \| gas card \| convenience store \| shop \| toilet \| entrance \| exit".  Field "Type" includes "transport facilities \| auto services \| gas filling \| life services". |
| Sinopec | Field "Name" includes "Sinopec" but not "card-selling \| recharge \| sales service centre \| gas card \| convenience store \| shop \| toilet \| entrance \| exit".  Field "Type" includes "transport facilities \| auto services \| gas filling \| life services". |
| WTP | / |
| DF | Field "Name" includes "DF \| livestock \| cow breeding \| milk source base".  Field "Type" includes "company". |

* The types of components and abbreviations are the same as those in Figure 1 of the main text.

† We apply the "str. contains "function in Python 3 for smart operations.

**References**

1. Chee, Y. E. An ecological perspective on the valuation of ecosystem services. *Biol. Conserv.* **120**, 549–565 (2004).

2. Chang, J. *et al.* Assessing the ecosystem services provided by urban green spaces along urban center-edge gradients. *Sci. Rep.* **7**, 1–9 (2017).

3. Liu, D. *et al.* Constructed wetlands as biofuel production systems. *Nat. Clim. Chang.* **2**, 190–194 (2012).

4. Chang, J. *et al.* Assessment of net ecosystem services of plastic greenhouse vegetable cultivation in China. *Ecol. Econ.* **70**, 740–748 (2011).

5. Fan, X. *et al.* Recoupling industrial dairy feedlots and industrial farmlands mitigates the environmental impacts of milk production in China. *Environ. Sci. Technol.* **52**, 3917–3925 (2018).
